# eDNA Metabarcoding Benchmarked towards Conventional Survey Methods in Amphibian Monitoring

**DOI:** 10.3390/ani12060763

**Published:** 2022-03-18

**Authors:** Anne Katrine Nørgaard Svenningsen, Cino Pertoldi, Dan Bruhn

**Affiliations:** 1 Department of Chemistry and Bioscience, Aalborg University, Fredrik Bajers Vej 7H, DK-9220 Aalborg, Denmark; cp@bio.aau.dk (C.P.); db@bio.aau.dk (D.B.); 2 Aalborg Zoo, Mølleparkvej 63, DK-9000 Aalborg, Denmark

**Keywords:** amphibian, biodiversity, conventional, eDNA, metabarcoding, conservation

## Abstract

**Simple Summary:**

Amphibian species are declining worldwide, and precise monitoring is key to ensuring timely protection and thereby ceasing deteriorating populations. Conventional monitoring methods are invasive, time-consuming, and dependent on expert knowledge. eDNA methods have been suggested as a replacement for or supplement to conventional survey methods. The present study assessed amphibian detection of conventional survey methods and eDNA metabarcoding in Danish lakes and ponds to address how the application of eDNA surveys can supplement the currently applied methodology. The study found eDNA metabarcoding to detect five out of six species detected through conventional methods. Furthermore, it is expected that the results in the present study reflect the time of sampling for the applied methods. The findings in the present study indicate that eDNA metabarcoding detects multiple Danish amphibian species and can produce knowledge on the occurrence and distribution for amphibian species. Implementing it as a supplement for conventional survey methods in nature monitoring will enable a higher frequency of monitoring and yield knowledge of species composition.

**Abstract:**

A keystone in protection work is accurate and thorough the monitoring of amphibian species, and the currently applied conventional survey methods are invasive, time-consuming, and dependent on expert knowledge. Research suggests that eDNA metabarcoding is a precise and cost-efficient method that could supplement the currently applied methods. The present study assessed the efficiency of conventional survey methods and eDNA metabarcoding in terms of species richness, the average number of detected species per site, the relative frequency of species occurrence, and the similarity of applied methods. The study found eDNA metabarcoding surveys to detect *Lissotriton vulgaris* (smooth newt), *Triturus cristatus* (great crested newt), *Rana arvalis* (moor frog), *Rana temporaria* (common frog), and *Bufo bufo* (common toad), as well as an average of 0.9 species per site, reflecting the species composition at the time of sampling in mid-July 2020. In addition to the species mentioned above, the conventional survey detected *Epidalea calamita* (natterjack toad) and an average of 1.7 species per site, reflecting the species composition at the time of sampling in early June 2020. The similarity between the methods applied in the present study was 27%, thus indicating a large number of unique observations of both eDNA metabarcoding and conventional surveys. The differences in detection can most likely be explained by the time of sampling, which was conducted a month apart. eDNA metabarcoding was efficient in detecting multiple amphibian species and produced unique observations that were not detected using conventional survey methods. Applying eDNA techniques as a supplement will most likely produce important knowledge on species distribution and presence, as well as enable more frequent monitoring due to cost efficiency and disturbance.

## 1. Introduction

The biodiversity crisis is observable worldwide, and the decline in amphibian species has been reported through the last century across the globe [1]. Loss of species can be linked to overexploitation, degradation and loss of habitats, pollution, invasion of alien species, and diseases [2]. According to the International Union for the Conservation of Nature (IUCN), an estimated 40% of the world’s amphibian species are endangered [3]. To cease the decline and protect species, thorough mapping of populations is necessary, yet the rate of this work has been influenced by challenges regarding, for example, amphibian lifecycles and the morphology of aquatic ecosystems. Commonly applied conventional methods in mapping amphibians constitute auditory and visual encounter surveys [4]. The methods are often difficult to carry out in practice due to varying accessibility to the whole perimeter of aquatic ecosystems, poor detection of elusive species, season and weather dependency, as well as speciation of tadpoles/larvae, which require expert knowledge and experience [5,6,7]. The success of the conservation of amphibian species relies on precise detection and mapping [2,8], and it is, therefore, important to evaluate the currently applied methodology against newly developed methods and current needs. 

DNA techniques have been suggested as a replacement or supplement to conventional survey methods to accommodate the shortcomings mentioned above in monitoring amphibian species [7,9,10]. Most amphibian species breed in still, fresh water in spring and summer [11], and this habitat offers a suitable medium for detecting environmental DNA (eDNA) secreted from the animals [12]. Research on the detection of amphibian species by eDNA techniques has been rapidly advancing since the progression of next-generation sequencing at the end of the 2000s, which reduced the cost and time consumption of the technique [13]. Researchers have found eDNA techniques to be equally or more efficient in detecting amphibian species compared to conventional survey methods [14,15,16], yet results are reliable on a number of abiotic and biotic factors which needs further research to be accounted for when conducting the methods [17,18]. The latest advances in DNA techniques have enabled the detection of multiple species in and across habitats through eDNA metabarcoding [9,19,20]. This method has been appointed as a promising tool in biodiversity monitoring, but the method has unresolved shortcomings such as risk of contamination, false negative and positive results, and challenges in determining abundance [14,21,22]. As with any novel method, there is a need for extensive research to ensure consistent workflow and reliable results [18,23]. To assess how eDNA metabarcoding could apply as a supplement to conventional methods, it is necessary to assess the methods’ ability to detect present species in varied freshwater environments with diverse species compositions [18,24]. Assays for both conventional and eDNA metabarcoding methods should target the expected species composition in an area, and it is essential to accumulate experience in a national context before implementing eDNA metabarcoding in nature monitoring.

The present study investigates conventional and eDNA metabarcoding survey methods for the detection of amphibian species. It evaluates efficiency concerning species richness, the average number of detected species, and the relative frequency of occurrence of amphibian species for conventional and eDNA metabarcoding survey methods. Furthermore, the similarity of observations between methods is assessed. In order to recommend the implementation of eDNA metabarcoding in nature monitoring, the method is expected to be efficient in detecting the species present at the time of sampling in terms of investigated parameters. The objectives of the present study are to (1) investigate conventional and eDNA metabarcoding survey methods’ detection of amphibian species richness and the average number of amphibian species per site of present and previous studies, (2) investigate the relative frequency of occurrence of amphibian species of conventional and eDNA metabarcoding surveys, and (3) compare detection similarities of species observation of the survey methods applied in the present study. Lastly, the advantages and disadvantages of applying conventional and eDNA metabarcoding survey methods in nature monitoring will be summarized.

## 2. Materials and Methods

**Figure 1:**
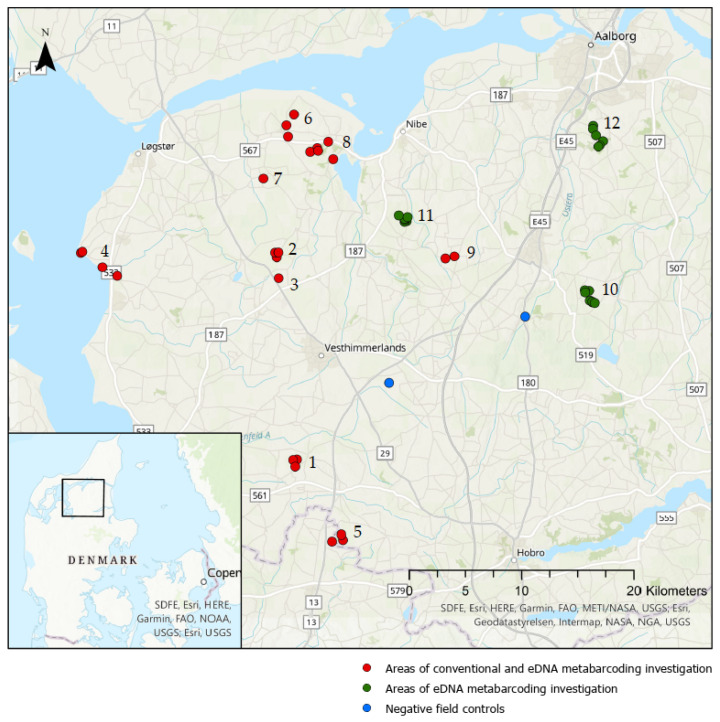
Overview of all investigated areas in Himmerland, Denmark. Red dots show areas where both eDNA metabarcoding and conventional survey methods were applied. Green dots show areas where eDNA metabarcoding was exclusively applied. Blue dots show areas of negative field controls for the eDNA metabarcoding survey. Numbers indicate an investigation area consisting of one to eight sites. Each dot represents a lake or pond.

To assess the objectives outlined above, field surveys were conducted in forty-seven lakes and ponds where previous observations (either from the national or municipal monitoring program) of amphibian species were available (Figure 1) (Supplementary A) [25,26]. In the present study, the field surveys constitute conventional surveys [4] and eDNA metabarcoding [13]. In areas 1–9, both conventional surveys and eDNA survey methods were applied, while areas 10–12 were investigated exclusively using eDNA metabarcoding. Species presence and absence detected by the applied methods are registered as incidence-based events. DNA traces of amphibians were filtered from the water on the sites of investigation and analyzed through eDNA metabarcoding to species level [27]. Two negative controls from streams were included in the study. The conventional survey was conducted in early June 2020, while eDNA for metabarcoding analysis was collected in mid-July 2020. When the study was initiated, the two methods were supposed to be conducted simultaneously. This was, however, not possible, thus yielding the applied study design.

### 2.1. Areas of Investigation

The search for amphibians was distributed in 12 different areas situated in Himmerland in Northern Jutland, Denmark. As mentioned above, the areas of investigation were chosen based on earlier studies (Supplementary A and B). For areas 10 and 11, a study by Neergaard [25] is used as a reference, and for all other areas, monitoring data from national or municipal authorities are applied [26]. All reference data arise from observations made from 2007 to 2014. The sites show a large degree of diversification in terms of morphology, some shallow and open, while others deep and weed-grown, which yields the potential of finding species with diverse habitat demands. Based on previous surveys, the presence of seven amphibian species could be expected in the areas of investigation: *Lissotriton vulgaris* (smooth newt), *Triturus cristatus* (great crested newt), *Pelobates fuscus* (common spade-foot toad), *Bufo bufo* (Common toad), *Epidalea calamita* (Natterjack toad), *Rana temporaria* (common frog) and *Rana arvalis* (moor frog) [25,26]. However, the occurrence of amphibians is subjected to large variations from year to year depending on, for example, weather conditions, and especially the occurrence of tadpoles in the water in July may vary.

### 2.2. Conventional Survey Methods 

The conventional survey was carried out according to the technical instruction for extensive amphibian surveys explained in this section [4]. The survey was carried out from 4 June to 13 June 2020, which corresponds to the recommended period of investigation for the species expected to be present. This procedure ensured that the method applied in the present study can be considered comparable to previous studies [25,26]. According to the technical instruction, each site (consisting of either a lake or pond) was divided into subsampling sites (10–20 sites depending on the size of the lake or pond) and, in total, searched for 30 min [4]. A dipnet (round opening 27.5 cm and mask size 2 mm) was used to search through the water column of the pond or lake with fast, smooth strokes of approximately 1.5 m/s in open water, on the bottom, and in elodeid vegetation. The subsampling sites were evenly distributed between the shore as well as open water and areas rich in vegetation. This method was applied to increase the probability of sampling all present species. Tadpoles or larvae, juvenile and adult individuals, and calls were speciated using Fog et al. [11], assessing morphological traits such as body shape and color and dentition. Due to great depths, overgrowth, or large mud layers at approximately half of the sites, it was not possible to conduct the search in accordance with the technical instruction (see Supplementary A for detailed information).

### 2.3. eDNA Sampling

The water samples for eDNA metabarcoding analysis were collected using sterile, disposable sampling kits from NatureMetrics (Egham, UK) following the provided manual [28]. The collection of water samples was carried out from 11 July to 16 July 2020, where the presence of species of interest is possible according to the technical instruction [4]. To avoid human contamination, gloves were worn at all times, and a disposable plastic cup placed on a shaft was used to retrieve water samples. Twenty subsamples of approximately 250 mL were collected from the whole perimeter of the lake or pond following the same principle as described in the technical instruction [4]. The subsamples were pooled in a sterile bag. A total of 50 mL of the well-mixed pooled sample was drawn up into a sterile syringe and pushed through a polyethersulfone filter (0.8 µm pore size) inside a plastic disk until clogged. The volume of filtered water was noted and ranged between 20 and 1500 mL, with an average of 432 mL (Supplementary C). Air was pushed through the filter to expel excess water, and hereafter a preservative solution was injected into the filter to stabilize the DNA and cease degradation, optimizing the yield. The filter was capped and kept at an ambient temperature and away from sunlight until the 5 August 2020, where it was shipped to NatureMetrics. Additionally, as controls, two samples were collected from streams to ensure no amphibian DNA would be contained herein (indicated with blue dots on Figure 1). These showed no traces of amphibian DNA above the determined threshold (Section 2.5).

### 2.4. Metabarcoding Laboratory and Library Analysis Procedure

The DNA extraction, PCR procedure and DNA sequencing were performed by NatureMetrics and are here described in brief. DNA extraction from forwarded filters was performed using DNeasy Blood and Tissue Kit (Qiagen GmbH, Hilden, Germany) and DNA was purified to remove PCR inhibitors using a DNeasy PowerClean Pro Cleanup Kit (Qiagen GmbH). The DNA extracts were quantified using Qubit dsDNA HS Assay Kit on a Qubit 3.0 fluorometer (Thermo Fisher Scientific, Waltham, MA, USA).

A hypervariable region of the 12S rRNA gene was amplified in a twostep PCR process. Firstly, 12 PCR replicates were performed on each sample using modified MiFISH primers [29] and were applied to target amphibians (under publication) and 12S-V5 primers to amplify vertebrate DNA [30,31]. The latter was applied on samples from sites 8.1, 8.2, 8.4, 11.1, 11.5, and 12.4 due to unsuccessful PCR, while the remaining samples were processed by applying the MiFish primers. PCR reactions were performed in a total volume of 10 μL. The vertebrate amplification mixture contained 1X DreamTaq PCR Master Mix (Thermo Fisher Scientific), 0.4 μM of each primer, 1 µL of template DNA, and PCR-grade water (Thermo Fisher Scientific). The amphibian amplification mixture consisted of 1X Phusion Green Mastermix (Thermo Fisher Scientific), an equimolar mix (0.3 μM) of three custom forward primers and the reverse primer (MiFish_UR), 1.5 mM of MgCl_2_ (Thermo Scientific), and 0.8 mg/mL of BSA (Thermo Fisher Scientific). PCR conditions consisted of: an initial denaturation at 95 °C for 2 min; 10 cycles of 20 s at 95 °C, a 30 s touchdown annealing step (−0.5 °C per cycle) starting at either 60 °C or 69 °C (vertebrate and amphibians, respectively), and 40 s at 72 °C; 35 cycles of 20 s at 95 °C, 30 s at 55 °C, and 40 s at 72 °C; and a final elongation step at 72 °C for 5 min. PCR-positive controls (i.e., a mock community with a known composition of non-native species) were included to verify sequence quality and PCR-negative controls (i.e., PCR-grade water) were included to detect potential cross-contamination. Amplification success was confirmed via gel electrophoresis and quantified using a Qubit^TM^ High-sensitivity kit (Thermo Fisher Scientific). All PCR replicates per sample were pooled and purified using Mag-Bind^®^ TotalPure NGS (Omega Bio-Tek Inc., Norcross, GA, USA) magnetic beads with a 0.8:1 (beads:DNA) ratio to remove primer dimer. A sequencing library was prepared from the purified amplicons using a combinational dual index approach, following Illumina’s 16S Metagenomic Sequencing Library Preparation protocol. Indexed PCR products were purified using Mag-Bind^®^ TotalPure NGS (Omega Bio-Tek Inc.) magnetic beads with a 1:1 (beads:DNA) ratio. Purified, indexed products were quantified using a Qubit dsDNA HS Assay Kit on a Qubit 3.0 fluorometer (Thermo Fisher Scientific), then pooled in equimolar concentration to create a final library. The final library was sized using a TapeStation D1000 ScreenTape System (Agilent) and normalized to 4 nM. The final library was loaded at 12 pM with a 10% PhiX control spike-in and sequenced with a MiSeq Reagent Kit v3 (600-cycles) on an Illumina MiSeq (Illumina, San Diego, CA, USA).

### 2.5. Bioinformatics

The processing of sequenced libraries was executed using the in-house workflow AmpProc5 version 5.1.0.beta2.12.0 in paired-end and VAR mode [32]. The workflow includes standardized quality filtering, merging of paired-end reads, removal of primers, zero-radius operational taxonomic unit (ZOTU) clustering [33], and chimera removal. The minimum expected amplicon length was set to 50 bp [15]. Taxonomic identification was performed using 12S MIDORI Unique metazoan vGB241 (2020-12) reference database [27]. A rarefaction curve of the ZOTUs showing flattening curves as the number of reads increases, indicating that the majority of the diversity of the samples has been sequenced, is included in Supplementary D [34]. Low-abundance ZOTUs were filtered at a 0.05% read threshold.

### 2.6. Statistical Analysis

The observation of amphibian species (either tadpole/larvae, adult, juvenile or call) through conventional surveys of both present and previous studies were registered in an incidence-based manner, where 1 indicates the presence of a species and 0 indicates the absence of a species. The observations of amphibian species through eDNA metabarcoding survey was registered in the same manner as for the conventional survey (incidence-based). The observation of species through methods applied in the present and previous studies yielded a maximum of seven registered amphibian species and between 0 and 4 species per site per method. The dataset for the conventional survey in the present study was constituted by twenty-four observations of present amphibian species distributed across twenty-seven investigation sites. The dataset for eDNA metabarcoding was constituted by forty-two observations of amphibian species distributed across forty-seven sites of investigation. The conventional surveys from previous studies were constituted by 111 observations distributed across forty-seven sites. Both presence and absence observations were registered in tables and applied for data analysis. All downstream data analysis and visualization was performed using RStudio version 1.4.1106 [35] with the VEGAN package version 2.5-7 [36] and ggplot2 version 3.2.1 [37], unless otherwise stated.

The *speccacum* function with the method *exact* and 100 permutations was applied to construct an accumulation curve establishing the expected species richness for all applied methods (conventional and eDNA metabarcoding in the present study and conventional surveys from previous studies [25,26]). The function calculates a standard deviation. The average number of detections of amphibian species as well as 95% confidence intervals were calculated for each method and a bar plot was created to visualize this. 

To further assess the conventional survey method and the eDNA metabarcoding method applied in the present study further analysis was performed. The species-specific detection was assessed using the relative frequency of occurrence, which was calculated for methods applied in the present study separately. The relative frequency of occurrence is defined as the number of species’ occurrences divided by the total number of occurrences of all species multiplied by 100. For visualization of this, a matrix plot of the relative frequency of occurrence was constructed using PAST version 4.05 [38]. 

To shed light on the similarity between the survey methods applied in the present study calculated using Sørensens Similarity Index ($S_{S}$) following Equation (1):

(1) $$S_{S}=\frac{\left(2\cdot a\right)}{\left(2\cdot a+b+c\right)}$$

where *a* is the number of observations the two methods share, *b* is the number of unique observations for method 1, and *c* is the number of unique observations for method 2 [39]. The index yields a number between 0 and 1, where 0 indicates no similarity and 1 indicates total similarity. This index is applied to assess the overlap of observations between the methods in order to evaluate detection on both a species level and on a general level.

## 3. Results

### 3.1. Species Richness and Average Detection

**Figure 2:**
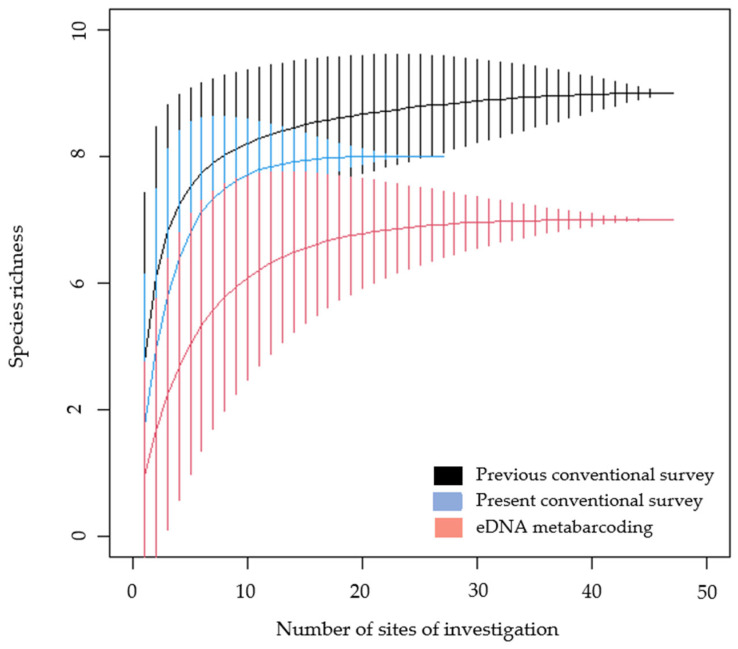
An accumulative curve of species richness detected by applied survey methods in the present and previous studies [25,26]. Vertical lines represent standard deviation. *n* = 27 for the conventional survey in the present study, while *n* = 47 for the conventional survey in previous studies and eDNA metabarcoding of the present study.

The present study detected six species by conventional methods, while the application of eDNA metabarcoding detected five species (Figure 2). The conventional survey in the present study detected *Epidalea calamita*, while this species was not detected using eDNA metabarcoding. The previous conventional surveys showed greater species richness compared to the present survey, with seven amphibian species in total [25,26]. Previous studies detected *Pelobates fuscus* [26], and this species was not detected in the present study regardless of the survey method. 

**Figure 3:**
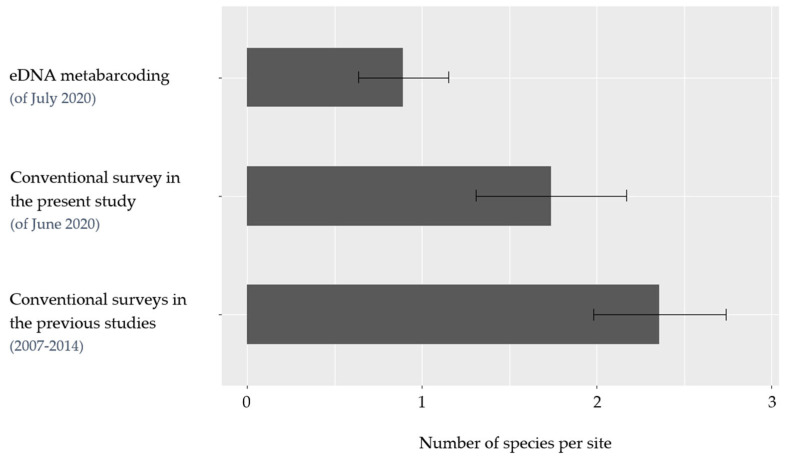
The average number of species detected per site per survey method. Bars indicate the average number of species detected per site (*x*-axis) by eDNA metabarcoding and conventional survey methods in the present and previous studies (*y*-axis) [25,26]. Lines with whiskers indicate 95% confidence interval.

The present study detected an average of 1.7 (ci 95%: 1.3, 2.2) species per site using conventional survey methods (Figure 3). Application of eDNA metabarcoding yielded an average of 0.9 (ci 95%: 0.6, 1.2) species per site. Previous studies detected an average of 2.4 (ci 95%: 2.0, 2.7) species per site [25,26]. 

### 3.2. Species-Specific Detection

**Figure 4:**
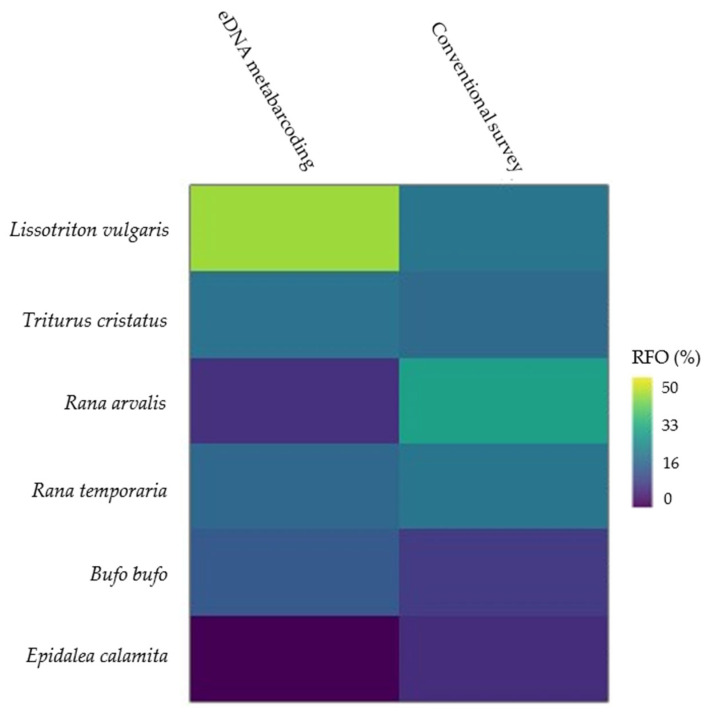
Matrix plot of the relative frequency of occurrence. A visualization of the relative frequency of occurrence (RFO) of amphibian species ranging from 0% (blue) to 50% (yellow) for each species and survey method applied in the present study.

The relative frequency of occurrence of *Lissotriton vulgaris* and *Bufo bufo* is the largest for eDNA metabarcoding. *Rana arvalis* has the lowest frequency of occurrence, and *E. calamita* was absent when applying this method (Figure 4 and Supplementary E). *R. arvalis* constitutes the highest relative frequency of occurrence in the conventional survey in the present study, while the lowest relative frequency of occurrence is found for *B. bufo* and *E. calamita.*

### 3.3. Similarity of Observations

**Figure 5:**
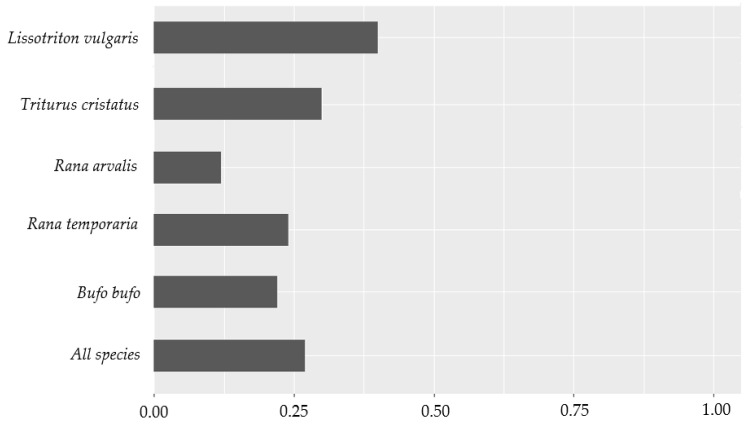
The similarity of observations between eDNA metabarcoding and conventional survey methods applied in the present study for all relevant species. The index value ranges from 0 to 1, where 0 indicates no similarity between the survey method and 1 indicates total similarity between the methods.

The similarity of species detection between survey methods applied in the present study shows that observations of *L. vulgaris* have the highest similarity (index value of 0.40). In contrast, observations of *R. arvalis* have the lowest similarity (index value of 0.12) (Figure 5 and Supplementary E). The similarity between the two methods was 0.27 for all species. There were no overlapping observations of *E. calamita*, and *P. fuscus* was not observed in the present study.

## 4. Discussion

### 4.1. Assessment of Efficiency

Based on previous studies [25,26], there were seven expected amphibian species in the areas of investigation: *Lissotriton vulgaris*, *Triturus cristatus*, *Rana arvalis*, *Rana temporaria*, *Bufo bufo*, *Epidalea calamita*, and *Pelobates fuscus*. The conventional survey in the present study revealed all but one expected species (*Pelobates fuscus)* and had an average detection of 1.7 species per site. eDNA metabarcoding detected five of seven expected species, leaving out *E. calamita* and *P. fuscus*, and had an average detection of 0.9 species per site. Seasonality is critical when investigating amphibians, and it is important to emphasize that the conventional survey and sampling for eDNA were conducted a month apart. Most amphibian species are present at the breeding grounds during spring and early summer, and the richness will be highest at this point [4]. Certain amphibian species such as *Rana* species and *E. calamita* migrate from the breeding grounds in mid-July. The average detection of species per site as well as species richness of eDNA metabarcoding in the present study reflects the species presence and composition at the time of sampling in mid-July, where presence will be naturally decreasing due to migration from the breeding grounds [11]. This trend is also reflected in the relative frequency of occurrence in the present study. The time of migration is known to be geographical and weather dependent, and it is likely that the *Rana* species in shallow and warm ponds have developed sufficiently to leave the breeding grounds before sample collection. In Denmark, during the spring of 2020, there was below-average rainfall, while several heat waves dominated in June [40], which could have contributed to elevated water temperatures, a large degree of evaporation from the sites and the rapid degradation of DNA in the environment between the time of conducting the conventional survey and sampling for eDNA [6,12]. eDNA metabarcoding has in other studies been shown to detect *Rana* species, and, despite weather conditions and time of sampling, both *R. arvalis* and *R. temporaria* were detected using the method in the present study [15,41]. However, the surveys conducted in the present study yielded an equal frequency of occurrence for *R. temporaria*, while there were great differences in frequency of occurrence for *R. arvalis.* In contrast to *R. arvalis*, which requires clean and warm water to breed, *R. temporaria* breeds in a broad range from warm, small, and open ponds to cool lakes with swampy areas. Depending on the type of breeding ground, the offspring of *R. temporaria* will develop at different paces. In shallow ponds, there will most likely occur a rapid development of the tadpoles, while they will remain in cool lakes for a longer period of time leaving sufficient amount of eDNA to be detected using this method in mid-July. *L. vulgaris* and *B. bufo* accounted for the largest share of frequency of occurrence for eDNA metabarcoding. Offspring of Salamandridae and *B. bufo* are expected to reside at the breeding grounds until August, which explains the relative frequency of occurrence for these species. 

The conventional survey detected *E. calamita* in both present and previous studies [25,26], but this species was not detected using eDNA metabarcoding. By conducting the search of *E. calamita* in the breeding season (from the middle of April until the beginning of July), the species should be easily located due to its loud and distinctive calls, yet tadpoles of the species can be challenging to speciate [11]. Unlike the other investigated species, *E. calamita* breed in very shallow and warm, temporary water bodies. Their offspring do not tolerate the presence of predators such as fish and newts, as well as competition from tadpoles of other species [11]. Offspring of *E. calamita* may have developed rapidly and migrated onto land before water sampling for eDNA metabarcoding analysis was conducted, explaining the lack of detection of the species. Detection of the species has been conducted by both eDNA qPCR [42,43] and metabarcoding [15]. To address whether the detection of *E. calamita* is possible with the assay applied in the present study, the inclusion of positive field controls is of importance.

### 4.2. A comparison with Historical Data

The conventional surveys in previous studies detected 2.4 species per site on average [25,26], which indicates a decrease of 0.7 species per site compared to the conventional survey in the present study. This difference could partly be attributed to a decline in amphibian species presence in Danish habitats recorded by the Danish Red List [44]. The previous studies stem from monitoring through 2007 to 2014, and the difference in average species detection is likely due to this decline. Furthermore, orthophotos revealed a substantial growth of woody vegetation since previous monitoring at certain sites (for example, 1.4, 5.1, 6.1, 8.4, 11.1, 11.3, and 11.4). This could contribute to a lower average detection of amphibians in the present study due to most of the species being reliant on freshwater environments with warm and relatively shallow water bodies with varying amounts of vegetation, yet not overgrown, in order to achieve breeding success [11]. This needs to be adjacent to amphibian resting grounds such as meadows or bogs that are non-fragmented. In Denmark, it is estimated that 60% of amphibian species have declined from 2010 to 2019, which also applies for *R. arvalis* and *R. temporaria* presently categorized as *nearly threatened*, while *E. calamita* is categorized as *threatened*, and *P. fuscus* is categorized as *vulnerable* [44]. The remaining three species investigated in the present study are categorized as *least concern.* This trend is predominant in all of the European Union, where an estimated 50% of amphibian populations are deteriorating [45]. This further emphasizes the need for efficient and thorough monitoring, enabling protection and ceasing of the current declining trends to avoid the possibly endemic extinction of amphibian species [3,46]. 

In a previous study [26], *P. fuscus* has been observed on the investigated sites (for example, 1.4, 2.3, 5.1, 5.2 and 6.1), yet the species was not detected in the present study regardless of the survey method. Both adults and tadpoles of *P. fuscus* are, to a great extent, eschewing human activity, and the males have faint calls that are best heard with special equipment at night [4,47]. Therefore, only experts can efficiently detect them in the field. The species might have disappeared from the investigated sites since previous studies conducted their field surveys in 2007 to 2014 [26], yet it is possible that the inspector of the present study overlooked individuals due to field surveys in early June and activity patterns of adults and tadpoles of *P. fuscus* [47]. *P. fuscus* was in 2019 estimated to be in decline in Denmark, where factors such as eutrophication of habitats, predation and limited ability to disperse are pointed out as plausible reasons for the decline [44]. A study showed the presence of *P. fuscus* using conventional survey methods and eDNA qPCR [16]. Positive field controls need to be included in the study design to investigate whether the presently applied assay for eDNA metabarcoding could detect *P. fuscus.* Furthermore, auditorial surveys should be included in the study design, as this is the most efficient conventional survey for detecting the occurrence of the species [4].

### 4.3. Similarity of Observations

The similarity of species detection between eDNA metabarcoding and conventional survey methods applied in the present study shows the highest similarity of observations for *L. vulgaris* (index value of 0.40) and *T. cristatus* (index value of 0.30). The similarity for observations of *R. arvalis* was lowest (index value of 0.12), while the similarities of *B. bufo* and *R. temporaria* were intermediate (index values of 0.22 and 0.24, respectively). The time of water sampling in the present study seems to favor Salamandridae over anuran species, which could be explained by the migration patterns of the species [11]. Investigations of sampling eDNA closer to the breeding season of anuran species (for example, early June) will most likely reveal a more evenly distributed pattern of species detection as shown by other studies [14,15,16]. Approximately 73% of the observations of the two methods are unique (in terms of a species at a given location at a given time), thus indicating that both methods contribute to uncovering the total species occurrence at the investigated sites and that the outcome of monitoring as of now will be more accurate when applying more than one survey method. The similarity between the applied methods underlines that amphibian species composition is changing throughout the season and that their lifecycle presents challenges that need to be considered when monitoring is conducted. Other studies indicate eDNA surveys methods to be equally or more efficient in detecting amphibian species compared to conventional survey methods [25,26], and lack of compliance between methods applied in the present study is most likely due to field survey protocols. Sampling protocols such as the amount of sampled and filtered water, the application ofsterilized equipment, and so forth should be considered [10]. Subsamples from the whole perimeter of each site were pooled to ensure the targeting of all possible present species. Sampling on some sites (for example, 1.4, 2.3, 7.1, and 9.2) only contained water samples from a delimited part of the water column due to overgrowth at the shoreline and water table. Furthermore, some sites had large amounts of suspended material in the water column, which is why filters of a larger pore size should be considered to ensure sufficient capture op eDNA during filtration [48]. Warm weather conditions in June 2020 facilitated the growth of, for example, algae, which possibly could contribute to a lower amount of filtrated water [40]. Furthermore, choices made in bioinformatic processing of the library could contribute to a mismatch between conventional and eDNA metabarcoding methods. The species investigated in the present study are, however, well described and therefore, the risk of misidentification is low. Human errors or contamination will most likely be the source if any. 

### 4.4. Survey Methods in the Practice of Nature Monitoring

In the planning of nature monitoring of amphibian species, it is essential to consider the methodology as to how the total species composition of an area is most efficiently and precisely assessed [18,49]. Expectations of the eDNA metabarcoding method need to be considered: does it replace conventional surveys, should it cover the species composition to the same extent as conventional methods, or is it implemented as a supplement to conventional surveys? It is important to contemplate that applying eDNA metabarcoding as a supplement presents specific issues such as the risk of contamination, false negative/positive, and sampling dependency [50]. In contrast to this, eDNA has the perks of being non-invasive, with a small impact on the environment compared to conventional methods [10]. Furthermore, studies suggest that eDNA metabarcoding is more cost-effective compared to conventional survey methods [10,51]. Conventional methods rely on species-specific sampling and have a certain degree of interobserver viability but have the potential to yield knowledge on habitat traits as well as species composition. Considering the number of experts of herpetology available for monitoring tasks within the season, it might be a wise choice to apply eDNA metabarcoding as a supplement to thorough baseline investigations. Implementing eDNA techniques as a supplement to conventional survey methods in nature monitoring over a period of time where both eDNA techniques and conventional survey methods are applied establishes a greater basis for comparison [52,53]. In this period, both methods should be analyzed in terms of efficiency in the detection of amphibian species and cost efficiency. Applying eDNA techniques will enable more frequent monitoring or monitoring of a greater area than what is possible with the currently applied methodology. This enables the focusing of conventional surveys on monitoring specific species or areas of particular interest and conducting protectional measures in monitored habitats based on species composition. The use of eDNA techniques would most likely contribute to the information on species presence and absence as well as on dispersal patterns and as the field advances and metabarcoding is refined, it will become more prevalent to replace conventional survey methods. Studies on shedding rates of DNA being specific to species, sex and age, DNA dispersal and degradation influenced by water movement, temperature, and UV radiation are important in developing robust eDNA assays [10]. Furthermore, studies on universal primers targeting vertebrates are ongoing and advancing and refining this field will possibly allow the implementation of eDNA metabarcoding covering multiple taxa of vertebrates, further contributing to simplify monitoring [54,55].

Ideally, in addition to conventional and eDNA survey methods, a model of habitat suitedness for all amphibian species should be developed and habitat traits registered. Other studies show a correlation between certain habitat traits and the presence of amphibian species, as well as the co-existence of species [56,57]. This could be traits such as depth, turbidity, aquatic and shore vegetation, surrounding areas (such as cultivated fields or meadows), and the presence of other aquatic vertebrates. To ensure future populations of amphibian species, dispersal between metapopulations is also essential, and corridors between habitats should also be incorporated in such a model. Robust models with several inputs have in other nature conservation projects been used to predict species migration and dispersal [58,59], which is valuable information in ensuring the cessation of the current decline in amphibian populations. Such a model will help the managers target their conventional monitoring to habitats suitable for species that, as of now, are difficult to detect by eDNA metabarcoding. Keeping the current limitations of the methods in mind, application eDNA techniques in areas with a baseline investigation on species composition could contribute with supplementary knowledge on species distribution and the co-existence of taxa, invasion, and migration. The frequency of monitoring could be increased or expanded to cover a greater area due to eDNA techniques being more cost-efficient than conventional survey methods. Before eDNA techniques offer a robust assay from sampling to results, there is still a need to conduct conventional surveys to ensure that the quality of nature monitoring is high and the conservation of amphibian species is conducted on a robust basis [18,24]. 

## 5. Conclusions

In conclusion, the eDNA metabarcoding survey detected *Lissotriton vulgaris*, *Triturus cristatus*, *Rana arvalis*, *Rana temporaria*, and *Bufo bufo* and an average of 0.9 species per site. These results reflect the expected species composition in mid-July when taking into account the weather conditions. Apart from the mentioned species, the conventional survey also detected *Epidalea calamita* and an average of 1.7 species per site, likewise reflecting the species composition in early June. The time of sampling was also reflected in the relative frequency of occurrence, where *R. arvalis* and *E. calamita* had greater relative frequency of occurrence in the conventional survey compared to the eDNA metabarcoding survey, which is most likely due to migration from the breeding grounds. The similarity of the eDNA metabarcoding and conventional methods of the present study was 27%, indicating a large degree of mismatch most likely due to sampling time as other studies find greater similarities. The amount of unique observation of both eDNA metabarcoding and conventional survey will accumulate to better uncover the total species composition if applied as complementary.

If eDNA metabarcoding is applied as a supplementary survey method to build upon a known species composition, it could provide information on species distribution and migration. By being more cost-effective than conventional surveys, the frequency of monitoring could be elevated or expanded to cover a greater area.

## Data Availability

Raw sequencing data are available at Supplementary Materials.

## Supplementary Materials

The following supporting information can be downloaded at: https://www.mdpi.com/article/10.3390/ani12060763/s1, Supplementary A: Description of areas of investigation; Supplementary B: Scheme for assessment of amphibian habitats; Supplementary C: Filtered water; Supplementary D: Rare fraction curve; Supplementary E: Additional results.
