# Supplementary material for: eDNA Metabarcoding Benchmarked towards Conventional Survey Methods in Amphibian Monitoring
Source: Animals (Basel). 2022 Mar 18;12(6):763. doi: 10.3390/ani12060763 (PMC8944553; doi:10.3390/ani12060763)
Supplement: Supplementary file 1 [file animals-12-00763-s001.zip › animals-1493411-supplementary.pdf]

animals

MDPI

Article

eDNA Metabarcoding Benchmarked towards Conventional Survey Methods in Amphibian Monitoring

Anne Katrine Nørgaard Svenningsen [1,*], Cino Pertoldi [1,2] and Dan Bruhn [1]

Supplementary A—Description of areas of investigation

The following contains a description of all areas of investigation in terms of morphology and usage. Each area consists of one or more lakes or ponds. The definition of a lake is an inland body of water between 1000 and 5000 m^2, while lakes larger than this will be described as large lakes (<5000 m^2). The definition of a pond is an inland body of water less than 1000 m^2. Descriptions of the lakes and ponds (e.g., vegetation and depth) are information gathered during fieldwork in June 2020 (see supplementary B).

Area 1 (56.720301, 9.480542)

Area 1 is a privately owned plantation primarily used for deforestation and hunting (personal communication with owner) and cultivated fields of conventional plant production. A pond (1.1) is situated inside the plantation, while one lake is found in the periphery of the plantation (1.2). The last pond is situated approximately 500 m from the plantation (1.3). The area was chosen for investigation due to findings through monitoring for the NOVANA program and municipal inspection [11]. Results from 2009 till 2014 showed the presence of *Lissotriton vulgaris, Triturus cristatus, Rana temporaria, Rana arvalis,* and *Pelobates fuscus*.

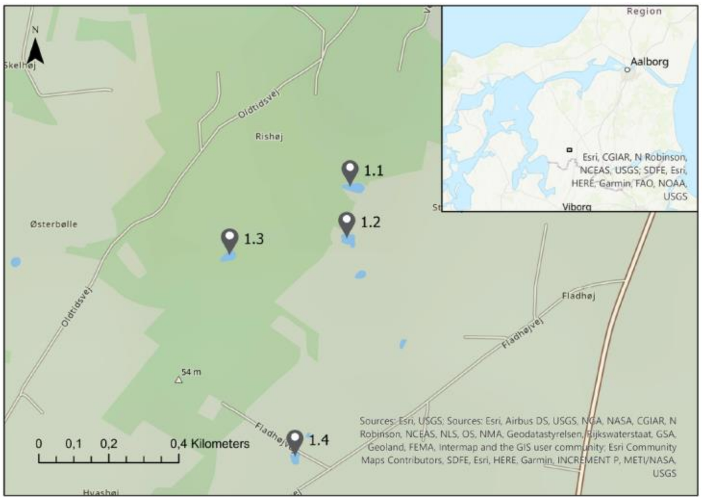

Figure A1. Area 1. The area has three investigated lakes and ponds, two of which are situated in or adjacent to the plantation and one approximately 500 meters from the plantation.

Site 1.1 is 1200 m^2, and approximately 70% is <1 m deep. The remaining part is >1.5 m deep. Trees and bushes cover 80% of the shoreline (e.g., Rhododendron and pine trees). Medium and high vegetation (16–50 cm) dominates the remaining part of the shoreline. The water is clear and with a large degree of benthic and submerged plants.

Site 1.2 is 1000 m^2, and more than 95 % is >1.5 m deep. Towards the south and west of the lake are areas covered by sphagnum. 60% of the shoreline is covered by trees and bushes, while 10% of the shoreline is adjacent to cultivated fields sloping towards the lake.

The remaining shoreline is covered by high vegetation (the category 26–50 cm) and is overgrown (>50 cm).

Site 1.3 is 670 m^2, where more than 30% is >1 m deep. Towards the south is a swampy area. 20% of the shoreline is overgrown (>50 cm), while medium to high vegetation (16–50 cm) covers the remaining.

Site 1.4 is 760 m^2 and has a large mud layer (between 50 and 100 cm), making it difficult to determine the depth. 99% of the shoreline is overgrown (>50 cm) and very difficult to access. Cultivated fields surround the pond, and it has experienced a large degree of growth of wooden vegetation since monitoring in 2009 (Danmarks Miljøportal, 2020).

Table A1. Overview of species findings by conventional methods in present and previous studies and eDNA metabarcoding. – indicates no findings. The previous study used as reference in this instance is Danmarks Miljøportal [11].

Site	Present Study		Previous Study
	**Conventional**	**eDNA Metabarcoding**	**Conventional**
1.1	*Lissotriton vulgaris*<br>*Triturus cristatus*	*Lissotriton vulgaris*<br>*Triturus cristatus*	*Triturus cristatus*<br>*Lissotriton vulgaris*<br>*Rana arvalis*<br>*Rana temporaria*
1.2	Anura sp.	*Lissotriton vulgaris*<br>*Rana temporaria*	*Triturus cristatus*<br>*Lissotriton vulgaris*
1.3	*Triturus cristatus*<br>*Lissotriton vulgaris*	*Lissotriton vulgaris*	*Triturus cristatus*<br>*Lissotriton vulgaris*<br>*Rana temporaria*
1.4	-	-	*Triturus cristatus*<br>*Rana temporaria*<br>*Pelobates fuscus*

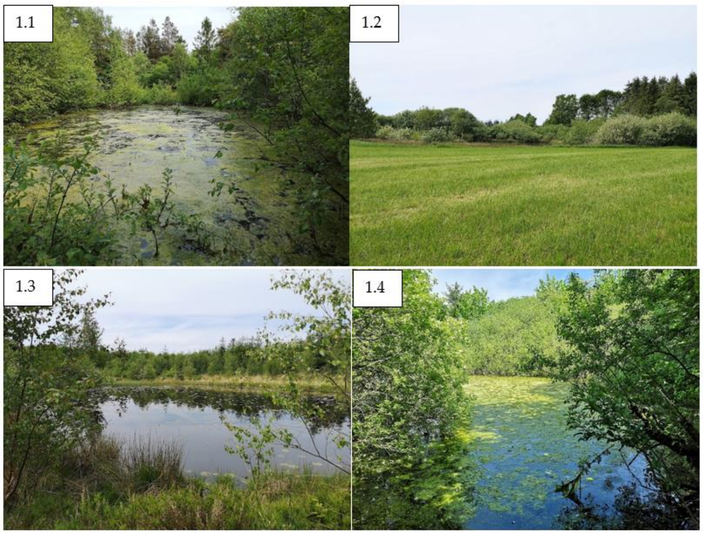

Figure A2. Photos of the four sites constituting area 1.

Area 2 (56.880112, 9.448668)

Area 2 is a large, protected area consisting of multiple nature types, including heathland and both large and small lakes. The whole area is 95.000 m^2 ha and is grazed by cattle and horses from April through October. The main lake is a clear, nutrition-poor lake at 22.000 m2. The area was chosen for investigation due to findings through monitoring for the NOVANA program and municipal inspection [11]. Results from 2009 till 2014 showed the presence of *T. cristatus*, *L. vulgaris*, *E. calamita*, *P. fuscus*, *B. bufo*, and *R. arvalis*.

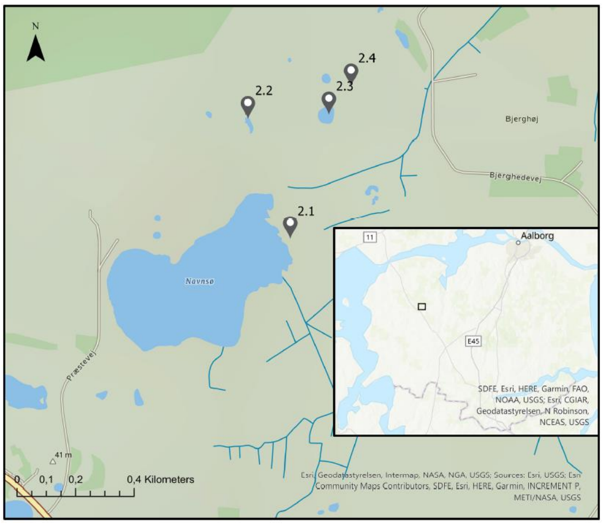

Figure A3. Area 2. The area has four investigated sites; one of which is a subarea of area 2 and two of which are smaller ponds. The last site is a narrow depening created by peat extraction.

2.1 is a subarea of the large lake Navnsø. It is situated in the northeastern part of the lake and is approximately 3500 m^2. Sand covers the bottom, the water is clear, and it has a depth of 35 cm. Medium to high vegetation (16–50 cm) dominates the shoreline, primarily bushes. Fish were observed at the site.

2.2 is 810 m^2 and is on average 50 cm deep. Furthermore, it has a large mud layer (50–70 cm), and approximately 20% of the shoreline is covered with reed swamp, while 50% has no vegetation and 30% has low to medium vegetation (1–25 cm).

2.3 is 850 m^2 and has since the monitoring period in 2009 developed into a transition mire. It is >1.5 m deep and has 5% uncovered water table. Due to unsafe conditions, it was not possible to search the entire area using conventional methods. >100 newly developed *R. arvalis* were observed when collecting water for eDNA.

2.4 is a subarea of a trough created by peat extraction. The site is 850 m^{2,} and 50% is >1.5 m deep, while 40% is approximately 1 m deep and 10 % is 50 cm deep. Reed swamp covers 70% of the area. Medium to high vegetation (16–50 cm) dominates the remaining part of the shoreline. In the western direction there are cultivated fields sloping towards the site.

Table A2. Overview of species findings by conventional methods in present and previous studies and eDNA metabarcoding. – indicates no findings. The previous study used as reference is in this instance is Danmarks Miljøportal [11].

Site	Present Study		Previous Study
	**Conventional**	**eDNA Metabarcoding**	**Conventional**
2.1	Bufonidae sp.<br>*Rana arvalis*	*Lissotriton vulgaris*<br>*Triturus cristatus*<br>*Rana temporaria*	*Rana arvalis*<br>*Lissotriton vulgaris*<br>*Bufo bufo*
2.2	*Rana arvalis*	*Bufo bufo*<br>*Rana temporaria*	*Rana arvalis*<br>*Epidalea calamita*
2.3	-	*Lissotriton vulgaris*<br>*Triturus cristatus*<br>*Rana temporaria*	*Triturus cristatus*<br>*Rana arvalis*<br>*Pelobates fuscus*
2.4	*Rana arvalis*	*Lissotriton vulgaris*<br>*Triturus cristatus*	*Triturus cristatus*<br>*Lissotriton vulgaris*<br>*Rana temporaria*

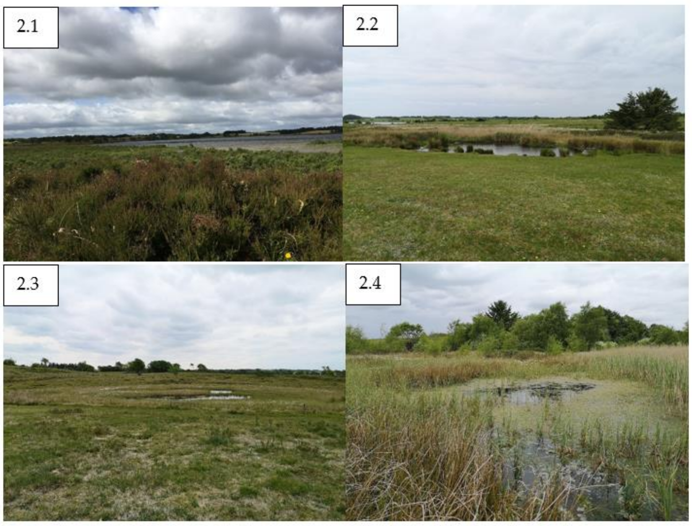

Figure A4. Photos of the four sites constituting area 2.

Area 3 (56.865362, 9.456003)

Area 3 consists of a lake situated on a cultivated field adjacent to a field grazed by horses. The area was formerly used as a gravel pit, but extraction of raw material ceased in 2013 and moved to a nearby location. Since 2013 lakes and ponds emerged in various places in the former gravel pit. This area was chosen for inspection due to findings of *E. calamita* and *L. vulgaris* in the nearby area during monitoring conducted by the Danish Environmental Protection Agency in 2013 [1].

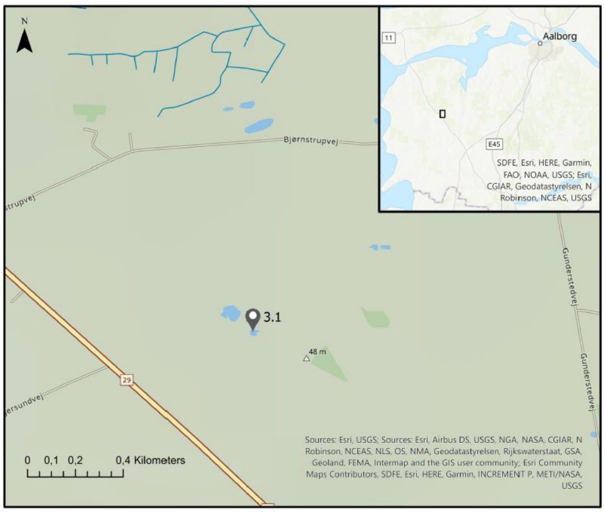

Figure A5. The area has one investigated site, which is a lake.

3.1 is 4600 m^2 and has a small mud layer (10 cm). The water has substantial amounts of suspended mud particles after periods with strong winds but is otherwise clear. It is approximately 75 cm deep and has a presence of Anseriformes. 80% of the shoreline has no or low vegetation (0–15 cm) and is cultivated, while the remaining 20% is adjacent to a windbreak.

Table A3. Overview of species findings by conventional methods in present and previous studies and eDNA metabarcoding. – indicates no findings. The previous study used as reference is in this instance is Danmarks Miljøportal [1].

Site	Present Study		Previous Study
	**Conventional**	**eDNA Metabarcoding**	**Conventional**
3.1	*Triturus cristatus*<br>*Epidalea calamita*<br>*Rana temporaria*	*Lissotriton vulgaris*	-

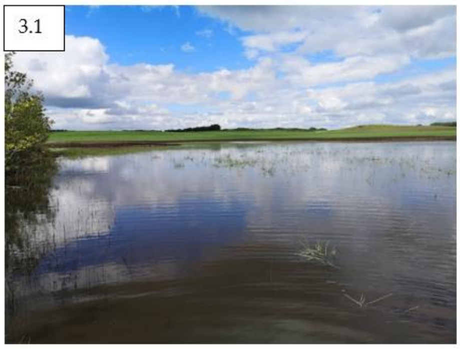

Figure A6. Photo of the one site constituting area 3.

Area 4 (56.881613, 9.181623)

Area 4 is situated by the Limfjord and consists of four sites of investigation. The area contains multiple nature types and is protected both by national and international legislation. Site 4.1, 4.2, and 4.3 are found in an Atlantic salt meadow close to the fjord (between 40 and 200 m), while site 4.4 is situated further inland. The area was chosen for investigation due to findings through monitoring for the NOVANA program and municipal inspection [11]. Results from 2009 till 2014 showed the presence of *E. calamita, R. arvalis,* and *T. cristatus*.

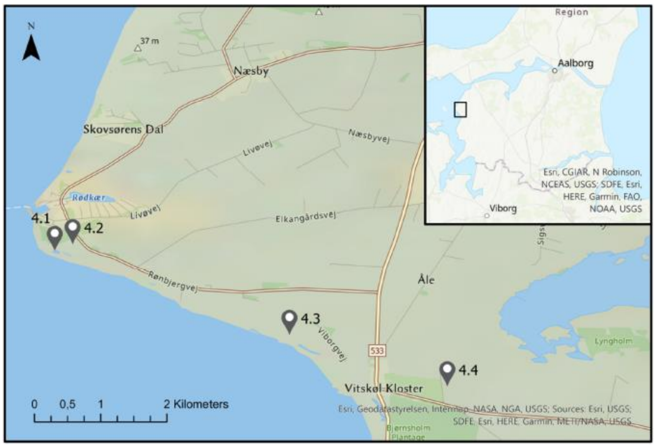

Figure A7. Area 4. The area has four investigated sites, which all are relatively shallow ponds.

4.1 has multiple small ponds and one large lake covering a total area of 4200 m^2. They are all shallow, with an average depth of 20 cm. Juncus effusus covers approximately 75% of the ponds. The shoreline is dominated by low to medium vegetation (1–25 cm). The ponds usually dry out in late July. The surrounding area shows signs of extensive grazing but no other management.

4.2 is 250 m^2 and has a depth of 75 cm. Typha sp. covers approximately 50% of the water body and shoreline. Medium to high vegetation (16–50 cm) dominates the

remaining part of the shoreline. The surrounding area corresponds to 4.1.

4.3 is 140 m^2 and has a depth of 75 cm. The water is blurry from suspended sand particles, and 90% of the shoreline is without vegetation. The area shows clear signs of intensive grazing by cattle, and they also seem to roam the pond.

4.4 is 380 m^2, and 50% of the pond has a depth of 75 cm, while the remaining have a depth >1m. High vegetation (25–50 cm) and overgrowth (>50 cm) dominates the shoreline. In close proximity are cultivated and grazed fields as well as a plantation.

Table A4. Overview of species findings by conventional methods in present and previous studies and eDNA metabarcoding. – indicates no findings. The previous study used as reference is in this instance is Danmarks Miljøportal [1].

Site	Present Study		Previous Study
	**Conventional**	**eDNA Metabarcoding**	**Conventional**
4.1	*Rana arvalis*<br>*Epidalea calamita*	*Lissotriton vulgaris*<br>*Triturus cristatus*<br>*Rana temporaria*	*Epidalea calamita*
4.2	*Rana arvalis*<br>*Epidalea calamita*	*Lissotriton vulgaris*	*Rana arvalis*
4.3	*Rana arvalis*<br>*Epidalea calamita*	*Lissotriton vulgaris*<br>*Rana temporaria*	-
4.4	*Lissotriton vulgaris*<br>*Triturus cristatus*	-	*Triturus cristatus*

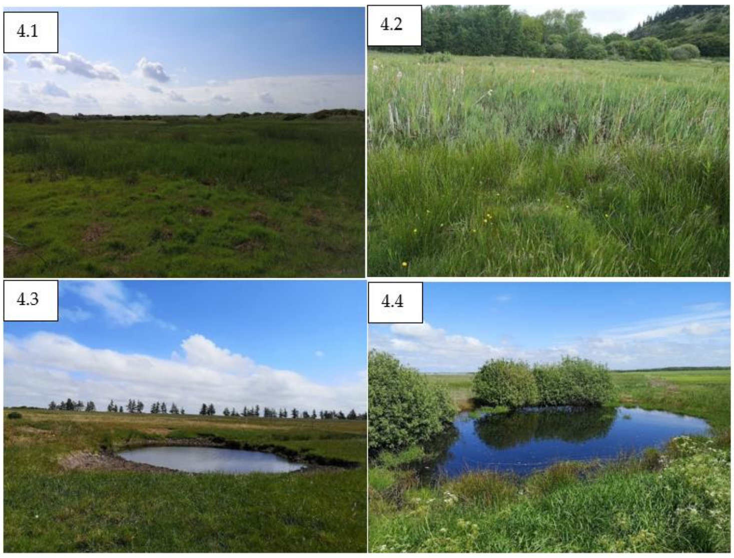

Figure A8. Photos of the four sites constituting area 4.

Area 5 (56.657344, 9.559132)

Area 5 consists of three sites, one of which (5.1) is situated in the open land, while the remaining two (5.2 and 5.3) are situated in a privately owned mixed forest and meadows. It is used for hunting, and areas around the lakes are otherwise not managed (personal communication with owner). The area was chosen for investigation due to findings through monitoring for the NOVANA program and municipal inspection [11]. Results from 2009 till 2014 showed the presence of *T. cristatus, L. vulgaris, R. temporaria, R. arvalis,* and *P. fuscus*.

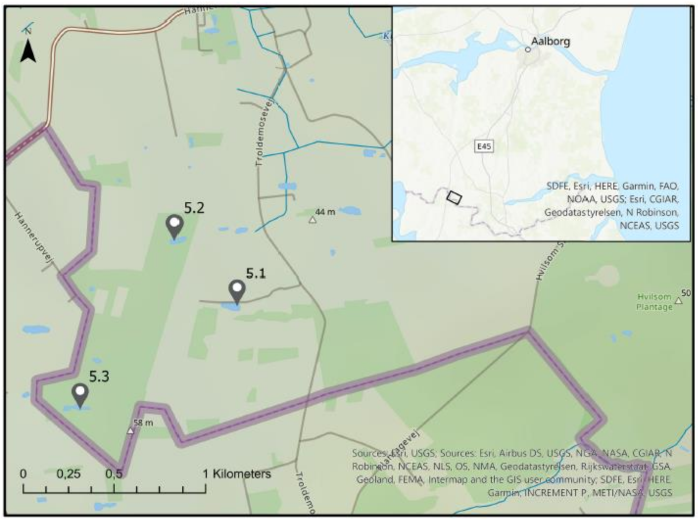

Figure A9. Area 5. The area of investigation has three sites, two of which are situated in a mixed forest and one of which is situated in the open land.

5.1 is 3500 m², and 50% of this area is less than 1 m deep, while the remaining is >1 m deep. Parts of the lake are currently developing into a transition mire, and plant material covers 80% of the water body (e.g., *Typha, Equisetum*, submerged plants). High vegetation (26–50 cm) and overgrowth (>50 cm) dominates the shoreline. 75% of the shoreline is close to cultivated fields sloping towards the lake.

5.2 is 2000 m² and surrounded by *Fagus sylvatica*, which shed large amounts of leaf material into the lake resulting in a large layer of organic material (up to 50 cm). The lake is up to 1.3 m deep, with 50% being relatively shallow. The shoreline has equal amounts of low, medium, and high vegetation (1–50 cm).

5.3 is 2500 m², and as for 5.2, this lake has large amounts of leaf material due to surrounding woody vegetation. 60% of the lake is >1 m deep, and low vegetation dominates the shoreline (1–15 cm). Not much sunlight reaches the lake due to tall trees.

Table A5. Overview of species findings by conventional methods in present and previous studies and eDNA metabarcoding. – indicates no findings. The previous study used as reference is in this instance is Danmarks Miljøportal [1].

Site	Present Study		Previous Study
	**Conventional**	**eDNA Metabarcoding**	**Conventional**
5.1	*Lissotriton vulgaris*	*Bufo bufo*<br>*Lissotriton vulgaris*<br>*Rana temporaria*	*Triturus cristatus*<br>*Rana temporaria*<br>*Rana arvalis*<br>*Pelobates fuscus*
5.2	*Rana arvalis*<br>*Rana temporaria*	*Bufo bufo*<br>*Lissotriton vulgaris*<br>*Triturus cristatus*	*Triturus cristatus*<br>*Lissotriton vulgaris*<br>*Rana temporaria*<br>*Pelobates fuscus*
5.3	-	-	*Triturus cristatus*<br>*Lissotriton vulgaris*<br>*Rana temporaria*<br>*Rana arvalis*<br>*Pelobates fuscus*

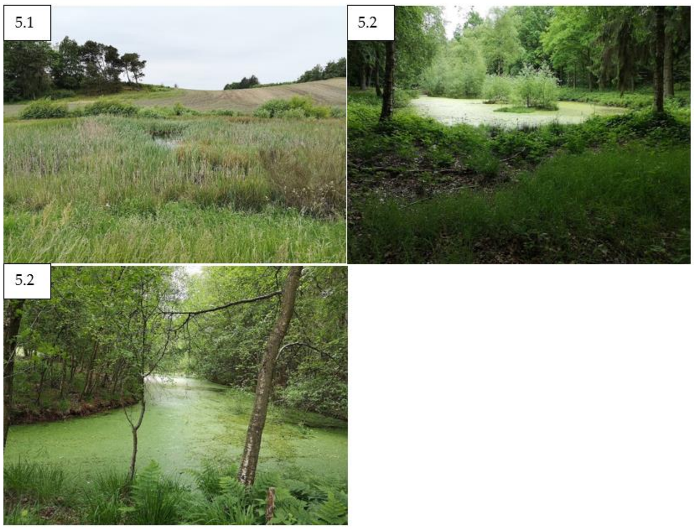

Figure A10. Photos of the three sites constituting area 5.

Area 6 (56.988343, 9.468561)

Area 6 consists of three sites; agricultural fields surround 6.1, 6.2 is on a field grazed by horses, and 6.3 is in a meadow. The area was chosen for investigation due to findings through monitoring for the NOVANA program and municipal inspection [11]. Results from 2009 till 2013 showed the presence of *Bufo bufo, T. cristatus, L. vulgaris, R. temporaria, R. arvalis,* and *P. fuscus*.

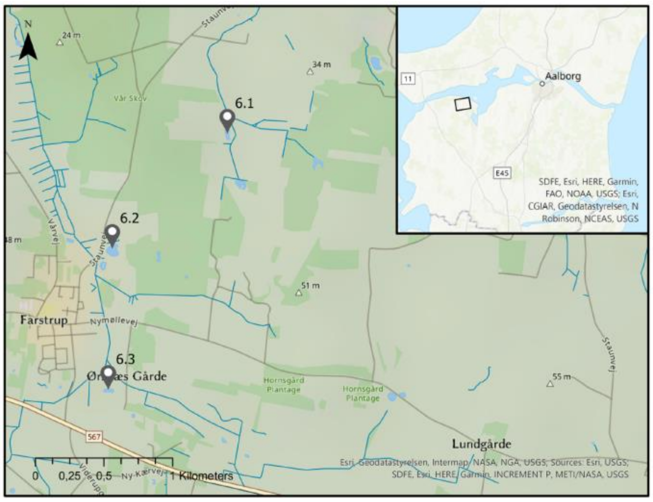

Figure A11. Area 6. The area of investigation has three sites, all situated in open land. 6.1 is surrounded by cultivated fields, while 6.2 is on a field grazed by horses. 6.3 is in a protected meadow.

6.1 is 1750 m^2, and 60% is <1 m deep while the remaining part is >1 m deep. Algae and small amounts of submerged plants dominate the water table. High vegetation (25–50 cm) and overgrowth (>50 cm) dominates the shoreline. The lake's proximity (up to 25 meters) is not cultivated, but agricultural fields surrounding the lake are sloping towards it. Furthermore a drain pipe with an inlet into the lake was observed. Fish and Anseriformes are present.

6.2 is 5000 m^2, and 20% is of is <1 m deep while the remaining part is >1 m deep. 50% of the shoreline is dominated by reed swamp with *Phragmites australis*, while the remaining part has no and low vegetation (0–15 cm). The surrounding field is grazed intensively by horses which have access to the lake. The surroundings are sloping towards the lake, and fish and Anseriformes are observed.

6.3 is 1600 m^2 and is <1.20 m deep. Vegetation such as *Equisetum, Typha, Juncus,* and submerged herbs covers approximately 50% of the water table. Medium to high vegetation (16–50 cm) dominates the shoreline, and the surrounding area is a meadow of the nature type 6410 (Molinia meadows on calcareous, peaty or clayey-siltladen soils (Molinion caeruleae) with dominant structures characteristics of 6410.

Table A6. Overview of species findings by conventional methods in present and previous studies and eDNA metabarcoding. – indicates no findings. The previous study used as reference is in this instance is Danmarks Miljøportal [1].

Site	Present Study		Previous Study
	Conventional	eDNA Metabarcoding	Conventional
6.1	*Bufo bufo*	*Triturus cristatus*	*Triturus cristatus*<br>*Lissotriton vulgaris*<br>*Bufo bufo*<br>*Rana temporaria*<br>*Rana arvalis*<br>*Pelobates fuscus*
6.2	*Bufo bufo*<br>*Rana temporaria*	-	*Rana temporaria*<br>*Rana arvalis*
6.3	*Bufo bufo*<br>*Rana arvalis*	*Triturus cristatus*<br>*Lissotriton vulgaris*	*Lissotriton vulgaris*<br>*Bufo bufo*<br>*Rana arvalis*

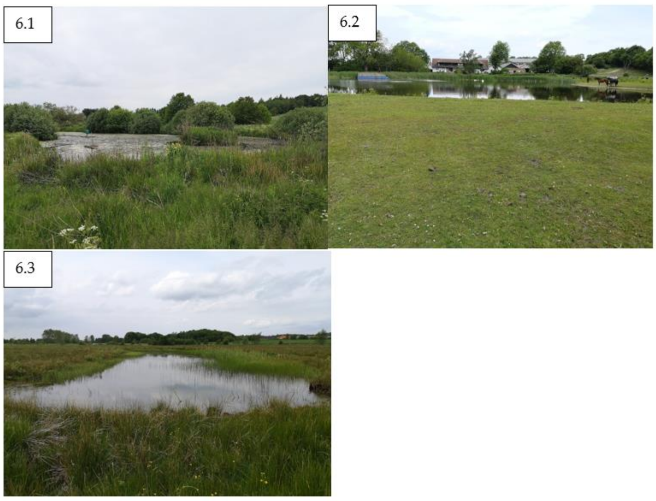

Figure A12. Photos of the three sites constituting area 6.

Area 7 (56.951357, 9.404843)

Area 7 consists of one site situated in a cultivated field. The area was chosen for investigation due to findings through monitoring for the NOVANA program and municipal inspection [1]. Results from 2009 till 2013 showed the presence of *Bufo bufo, L. vulgaris, R. temporaria, R. arvalis,* and *P. fuscus.*

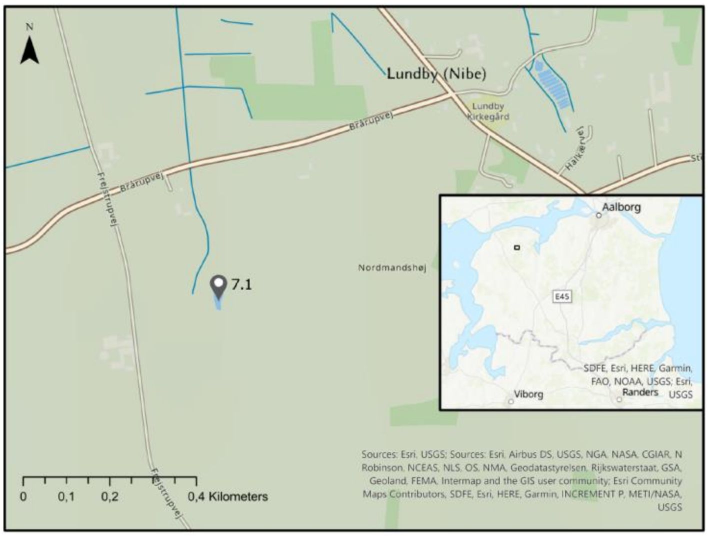

Figure A13. Area 7. The area of investigation has one site situated on a cultivated field.

7.1 is 760 m² and has a large layer of mud and organic material (<70 cm), making it difficult to determine the depth. Vegetation such as *Equisetum, Typha,* and *Juncus* covers 90% of the water table, and steep bluffs enclose the southern half of the pond. These structures make it difficult to search the site for amphibians.

Table A7. Overview of species findings by conventional methods in present and previous studies and eDNA metabarcoding. – indicates no findings. The previous study used as reference is in this instance is Danmarks Miljøportal [1].

Site	Present Study		Previous Study
	Conventional	eDNA Metabarcoding	Conventional
7.1	-	*Lissotriton vulgaris*<br>*Bufo bufo*	*Lissotriton vulgaris*<br>*Bufo bufo*<br>*Rana temporaria*<br>*Rana arvalis*<br>*Pelobates fuscus*

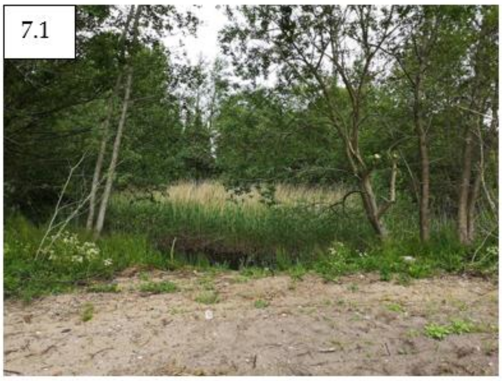

Figure A14. Photo of the one site constituting area 7.

Area 8 (56.969963, 9.513487)

Area 8 consists of five versatile sites (see detailed detribtion below), but the site has no intentive management in near proximity except 8.4 which has agricultural fields sorrounding it. The area was chosen for investigation due to findings through monitoring for

the NOVANA program and municipal inspection [1]. Results from 2009 till 2014 showed presence of *T. cristatus, R. temporaria, R. arvalis* and *P. fuscus.*

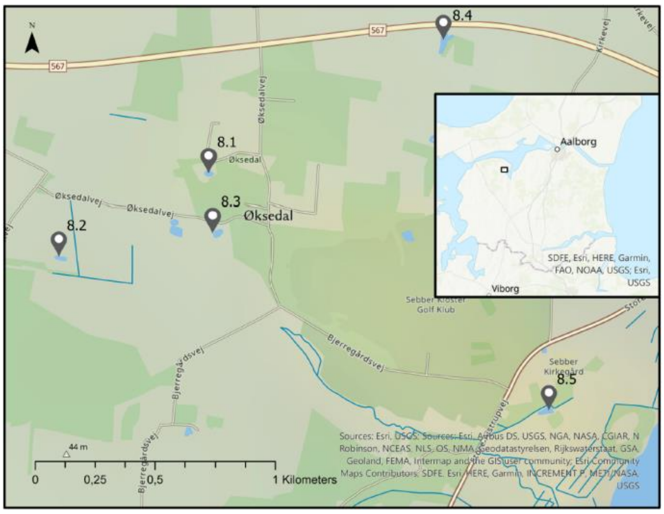

Figure A15. Area 8. 5 sites constitute the area of investigation, and they are diverse in terms of morphology and surroundings.

8.1 is 1000 m^2, and 30% is <1m deep, while the remaining part is >1 m deep. Overgrowth (primarily *Betula* and *Pinus*) dominates the shoreline, while a minor part has no or low vegetation (0–15 cm). The water table has small amounts of submerged herbs. In the surrounding area there is a forest and cultivated fields sloping towards the site.

8.2 is 1300 m^2, and 60% is <1m deep, while the remaining part is >1 m deep. Algae and Iris dominate the water table, and low vegetation (1–15 cm) dominates the shorelines. The site is in a meadow grazed extensively by horses. It does not show signs of management besides grazing.

8.3 is a large wetland area of which 3800 m^2 were searched for amphibians. The area was < 1 deep and covered by vegetation such as *Juncus*, Gramineae, and Cyperaceae. The site is situated in an open forest and is periodically underwater. Therefore, overgrowth (>50 cm) of woody vegetation dominates the shoreline. The area shows signs of natural grazing and hunting.

8.4 is a large lake of which 2200 m^2 was searched for amphibians. 20% of the area is <1 m deep, while the remaining part is >1 m deep. Buffer strips surround the area to avoid disturbance from agricultural activities in near proximity. However, towards north and east are cultivated fields that slope towards the lake. Medium and high vegetation (16–50 cm), as well as overgrowth (>50 cm), dominates the shoreline.

8.5 is 1100 m^2, and 20% of the area is <1 m deep, while the remaining part is >1 m deep. There is a small layer of organic material covering the bottom (20 cm). *Lemna, Iris pseudacorus,* and *Phragmites australis* cover 95% of the water table, which also dominates the shoreline. Surrounding the site are cut and fertilized grass as well as old trees.

Table A8. Overview of species findings by conventional methods in present and previous studies and eDNA metabarcoding. – indicates no findings. The previous study used as reference is in this instance is Danmarks Miljøportal [1].

Site	Present Study		Previous Study
	**Conventional**	**eDNA Metabarcoding**	**Conventional**
8.1	*Lissotriton vulgaris*<br>*Triturus cristatus*	*Lissotriton vulgaris*<br>*Rana temporaria*	*Triturus cristatus*<br>*Rana temporaria*
8.2	*Lissotriton vulgaris*<br>*Triturus cristatus*	*Lissotriton vulgaris*	*Triturus cristatus*<br>*Rana arvalis*

	Rana arvalis<br>*Rana temporaria*		*Rana temporaria*
8.3	*Lissotriton vulgaris*<br>*Triturus cristatus*<br>*Rana temporaria*	*Lissotriton vulgaris*	*Rana arvalis*<br>*Rana temporaria*
8.4	*Triturus cristatus*<br>*Rana arvalis*<br>*Rana temporaria*<br>*Bufo bufo*	-	*Triturus cristatus*<br>*Rana arvalis*<br>*Rana temporaria*
8.5	*Rana temporaria*<br>*Rana arvalis*	*Lissotriton vulgaris*<br>*Rana temporaria*	Amphibia sp.

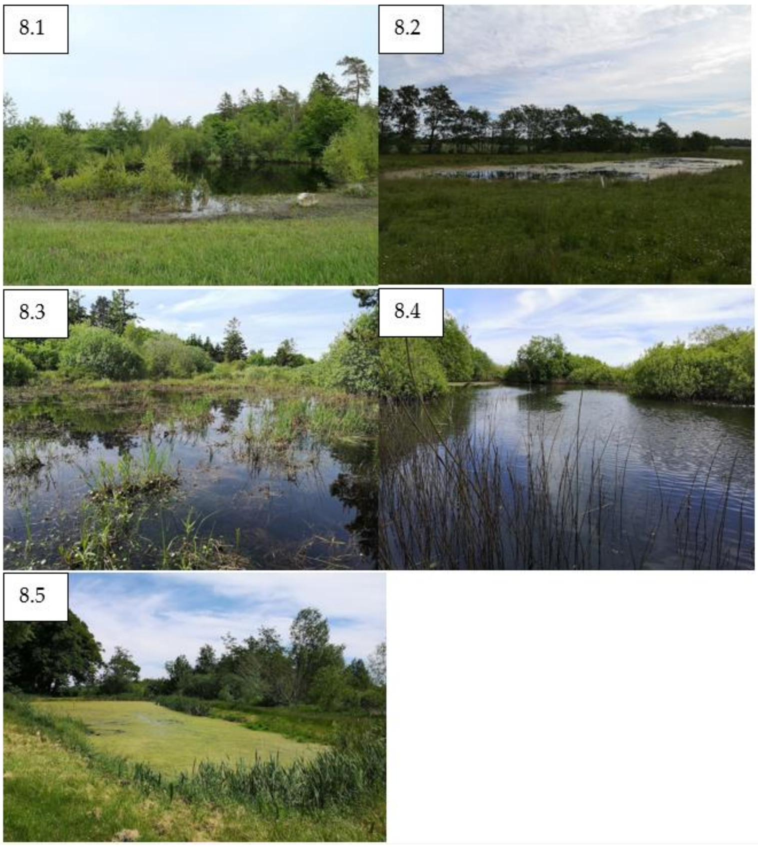

Figure A16. Photos of the five sites constituting area 8.

Area 9 (56.881594, 9.712750)

Area 9 consists of two sites both of which are situated in open land with cultivized field in near proximity. The area was chosen for investigation due to findings through monitoring for the NOVANA program and municipal inspection [1]. Results from 2009 till 2014 showed presence of *T. cristatus, L. vulgaris, R. arvalis* and *P. fuscus*.

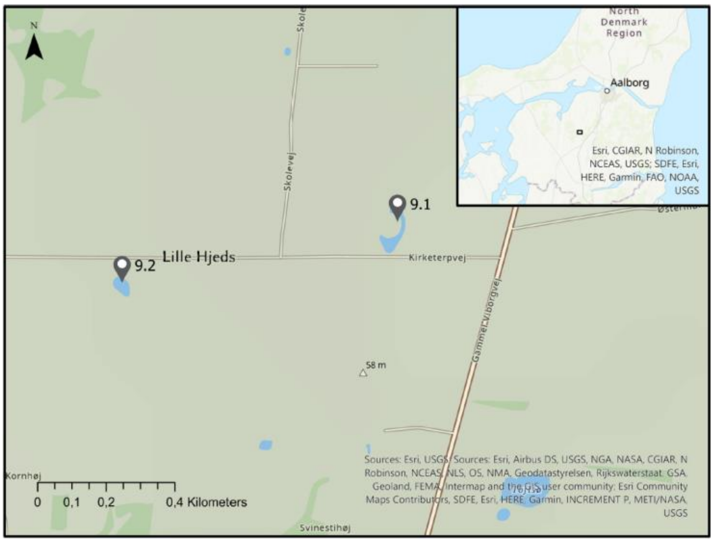

Figure A17. Area 9. The area of investigation consists of two sites, both surrounded by agricultural fields and near buildings.

9.1 is a large wetland area of which 4000 m^2 was searched for amphibians. The site is 0.2 m deep, and *Equisetum, Typha,* and woody vegetation covers 90% of the water table, making it challenging to investigate amphibian presence. Overgrowth (>50 cm) dominates the shoreline, and cultivated fields and a road surrounds the lake. At the time of water collection for eDNA metabarcoding, a large area was almost dried out.

9.2 is 1800 m^2, and 30% is <1m deep, while the remaining part is >1 m deep. *Equisetum, Typha,* and woody vegetation dominate the water table. High (26–50 cm) and overgrowth (>50 cm) dominates the shoreline. Cultivated fields and buildings were situated near the lake.

Table A9. Overview of species findings by conventional methods in present and previous studies and eDNA metabarcoding. – indicates no findings. The previous study used as reference is in this instance is Danmarks Miljøportal [1].

Site	Present Study		Previous Study
	Conventional	eDNA Metabarcoding	Conventional
9.1	*Lissotriton vulgaris*<br>*Rana arvalis*<br>*Rana temporaria*	-	*Triturus cristatus*<br>*Rana arvalis*
9.2	*Lissotriton vulgaris*<br>*Triturus cristatus*<br>*Rana arvalis*<br>*Rana temporaria*	*Lissotriton vulgaris*<br>*Rana temporaria*	*Lissotriton vulgaris*<br>*Triturus cristatus*<br>*Rana arvalis*<br>*Pelobates fuscus*

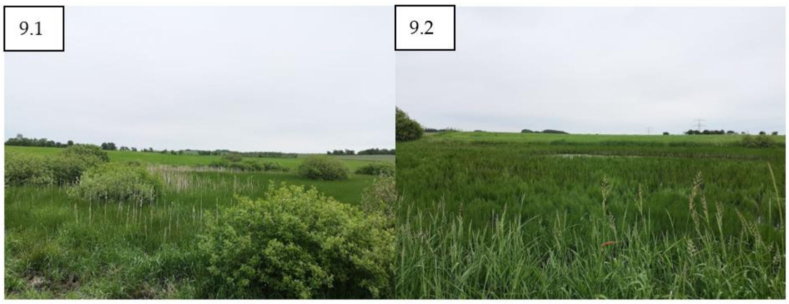

Figure A18. Photos of the two sites constituting area 9.

Area 10 (56.848871, 9.909558)

Area 10 consists of eight versatile sites; some close to agriculture and golf courses and some in protected areas. The area was chosen for investigation due to findings through investigations required by local municipal authorities conducted by Amphi-Consult [2]. Results from 2012 showed the presence of *B. bufo, L. vulgaris, T. cristatus, R. temporaria,* and *R. arvalis.*

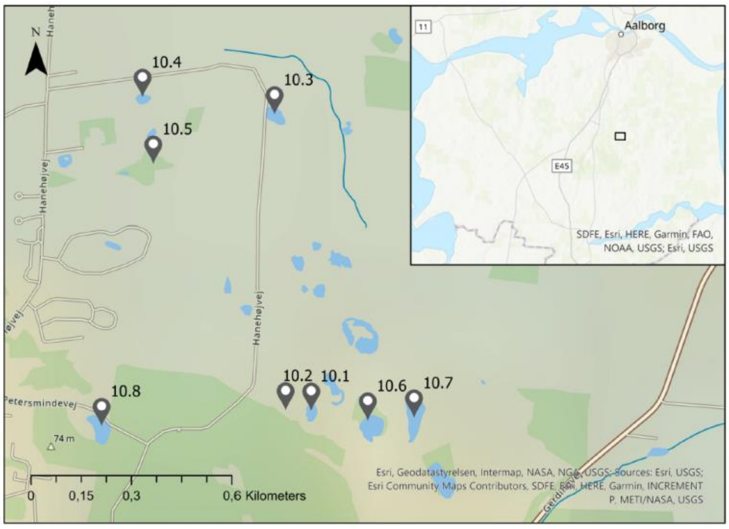

Figure A19. Area 10. The area of investigation consists of two sites, both surrounded by agricultural fields and close to buildings.

10.1 is 1200 m², and 90% is >1 m deep. Small amounts of submerged vegetation cover the water table, and high vegetation (26–50 cm) and overgrowth by trees and *Typha* dominate the shoreline. A golf course surrounds and slopes towards the site.

10.2 is 500 m² and >1.5 m deep. The banks are very steep, and the water is unclear and brownish. Trees surround the site and towards north is a cultivated field sloping towards the site. Large amounts of dead wood are lying in the water, and *Lemna* covers the water table.

10.3 is 1950 m², and 95% is >1 m deep. Submerged vegetation covers 30% of the water table, and high vegetation (25–50 cm) and overgrowth (<50 cm) dominates the shoreline. Cultivated fields surround the site.

10.4 is 1100 m² and 0.5 m deep. 95% of the water table is covered by aquatic vegetation, and low to medium vegetation (1–40 cm) dominates the shoreline. The site is in a field grazed extensively by horses.

10.5 is a part of a larger wetland area, and 500 m² is searched for amphibians. It is >1.2 m deep, and trees surround the site. Large amounts of dead wood lie in the water, and *Lemna* covers 50% of the water table. The site is in a peat surrounded by grassland.

10.6 is 3700 m², and 95% is >1 m deep. Small amounts of aquatic vegetation cover the water table, and large trees dominate the shoreline. In combination, these characteristics made the collection of water from the whole perimeter of the water column impossible. A golf course is close to and sloping towards the site.

10.7 is 3900 m², and 50% is <1 m deep. Aquatic vegetation covers the 40% water table, and large trees dominate the shoreline. In combination, these characteristics made the collection of water from the whole perimeter of the water column impossible. A golf course is close to and sloping towards the site.

10.8 is 3400 m², and 60% is <1 m deep. Aquatic plants cover 80% of the water table, and trees, bushes, and Juncaceae dominate the shoreline. In close proximity to the site are forests and meadows.

Table A10. Overview of species findings by conventional methods in previous study and eDNA metabarcoding. – indicates no findings. The previous study used as reference is in this instance is Neergaard [2].

Site	Present Study	Previous Study
	**eDNA Metabarcoding**	**Conventional**
10.1	-	*Lissotriton vulgaris*<br>*Triturus cristatus*<br>*Rana arvalis*<br>*Rana temporaria*<br>*Bufo bufo*
10.2	*Lissotriton vulgaris*<br>*Rana temporaria*	*Rana sp.*<br>*Lissotriton vulgaris*<br>*Triturus cristatus*
10.3	*Lissotriton vulgaris*<br>*Triturus cristatus*	*Lissotriton vulgaris*<br>*Triturus cristatus*
10.4	*Lissotriton vulgaris*<br>*Triturus cristatus*	*Lissotriton vulgaris*<br>*Triturus cristatus*<br>*Rana arvalis*<br>*Rana temporaria*
10.5	*Lissotriton vulgaris*<br>*Bufo bufo*	*Lissotriton vulgaris*<br>*Rana arvalis*<br>*Rana temporaria*
10.6	*Lissotriton vulgaris*<br>*Rana temporaria*<br>*Bufo bufo*	*Lissotriton vulgaris*<br>*Triturus cristatus*<br>*Rana arvalis*<br>*Rana temporaria*
10.7	*Lissotriton vulgaris*<br>*Rana temporaria*	*Rana arvalis*<br>*Bufo bufo*
10.8	*Lissotriton vulgaris*<br>*Triturus cristatus*<br>*Rana temporaria*	*Rana arvalis*<br>*Rana temporaria*<br>*Bufo bufo*

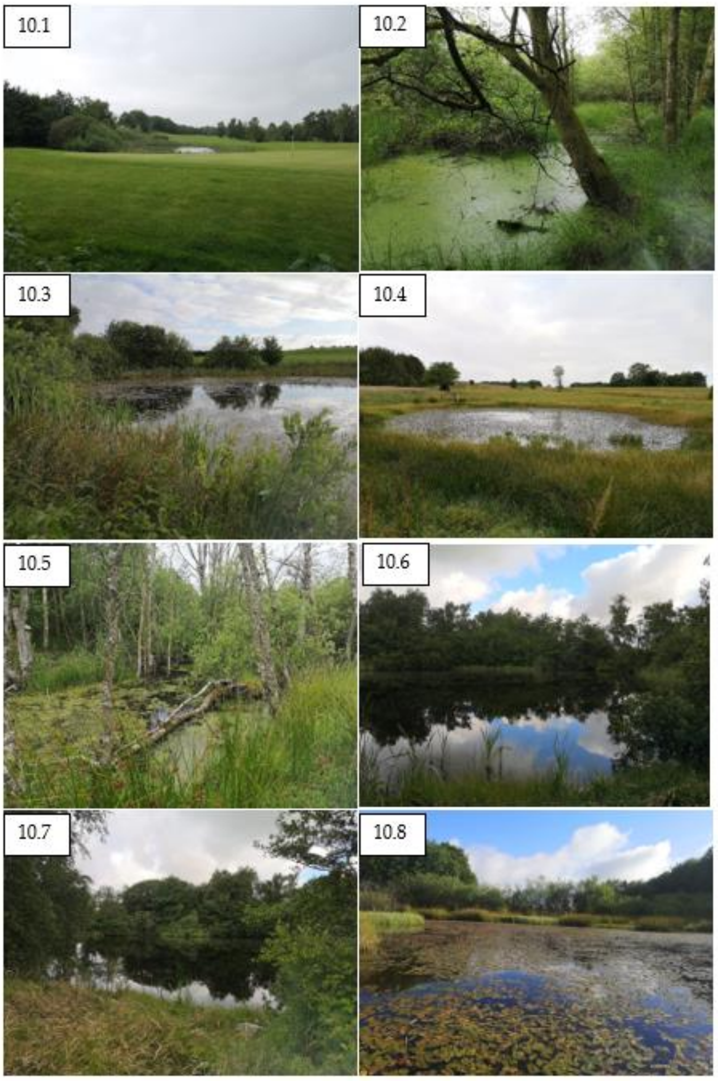

Figure A20. Photos of the eight sites constituting area 10.

Area 11 (56.913024, 9.638708)

Area 11 consists of six sites, five of which are in a plantation and while one in a grazed field. The privately-owned plantation is primarily used for deforestation and hunting (personal communication with the owner). The area was chosen for investigation due to findings through investigations required by local municipal authorities conducted by AmphiConsult [2]. Results from 2012 showed the presence of *B. bufo, L. vulgaris, T. cristatus, R. temporaria,* and *R. arvalis.*

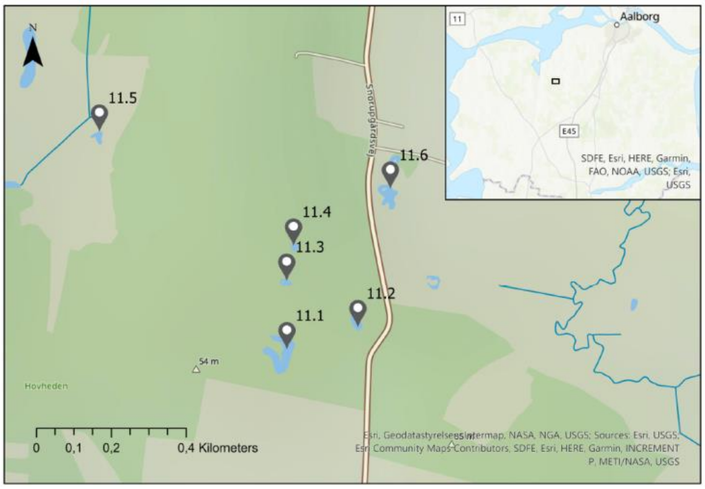

Figure A21. Area 11. The area of investigation consists of two sites, both surrounded by agricultural fields and close to buildings.

11.1 is 680 m² and <1.3 m deep. Submerged plants cover 80% of the water table, and large trees and Juncaceae cover the shoreline. Both *L. vulgaris* and *T. cristatus* was observed at the site.

11.2 is part of a large, deep lake, and 600 m² was searched for amphibians. 50% of the site is <1 m deep with some aquatic vegetation. Large trees and bushes dominate the shoreline, making it difficult to collect water samples from the whole perimeter of the water column.

11.3 is 450 m² and <1 m deep. Aquatic vegetation covers 60% of the water table, and trees, bushes, and Poaceae dominates the shoreline.

11.4 is 300 m² and <1 m deep. The water appeared brownish, and no to little aquatic vegetation was observed. Trees, bushes, and Poaceae dominate the shoreline. Forest and meadow surround the site.

11.5 is a part of a large wetland area of which 600 m² was searched for amphibians. *Typha* covered the whole water table making it difficult to assess the depth of the site and collect water from the whole perimeter of the water column. Adults of *R. temporaria* were observed at the site.

11.6 is 230 m². *Typha* cover the whole water table making it difficult to assess the depth of the site and collect water from the whole perimeter of the water column. Trees and bushes dominated the shoreline.

Table A11. Overview of species findings by conventional methods in previous study and eDNA metabarcoding. – indicates no findings. The previous study used as reference is in this instance is Neergaard [2].

Site	Present Study	Previous Study
	eDNA Metabarcoding	Conventional
11.1	*Lissotriton vulgaris*<br>*Rana temporaria*<br>*Bufo bufo*	*Lissotriton vulgaris*<br>*Rana arvalis*<br>*Rana temporaria*<br>*Bufo bufo*
11.2	-	*Lissotriton vulgaris*<br>*Rana arvalis*
11.3	*Rana temporaria*	*Lissotriton vulgaris*<br>*Rana arvalis*
11.4	*Lissotriton vulgaris*<br>*Triturus cristatus*	*Lissotriton vulgaris*<br>*Rana arvalis*

11.5	-	*Lissotriton vulgaris*<br>*Rana arvalis*<br>*Rana temporaria*<br>*Bufo bufo*
11.6	*Lissotriton vulgaris*<br>*Triturus cristatus*<br>*Rana temporaria*	*Lissotriton vulgaris*<br>*Triturus cristatus*<br>*Rana arvalis*<br>*Rana temporaria*

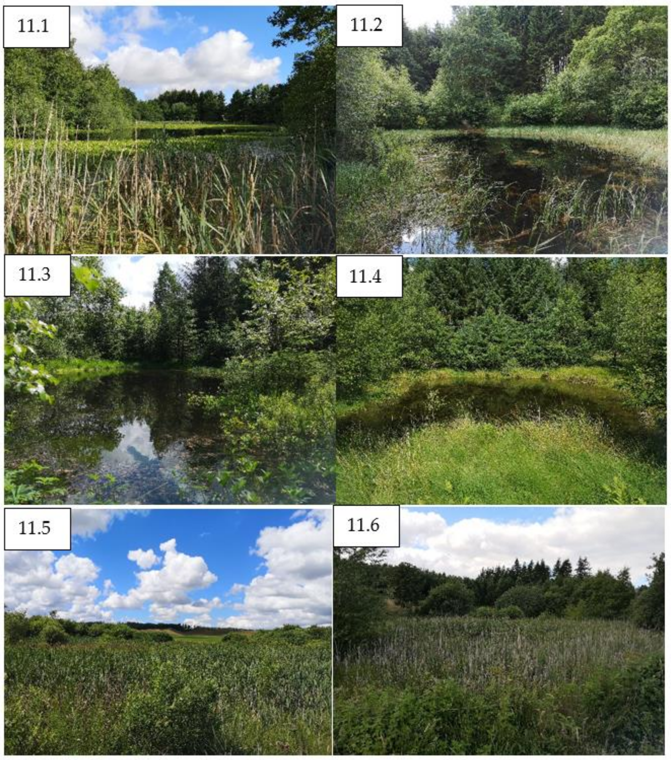

Figure A22. Photos of the six sites constituting area 11.

Area 12 (56.973567, 9.922598)

Area 12 consists of six sites; four (12.1–3 and 12.6) of which are situated in a protected area with several nature types such as heathlands, meadows and grasslands. These nature types are protected by the Danish Protection of Nature Act §3. 12.4 is situated in a forest area and 12.5 is situated in a small village. The area was chosen for investigation due to findings through monitoring for the NOVANA program and municipal inspection [1]. Results from 2009 till 2014 showed presence of *B. bufo, T. cristatus, L. vulgaris, R. arvalis* and *R. temporaria*.

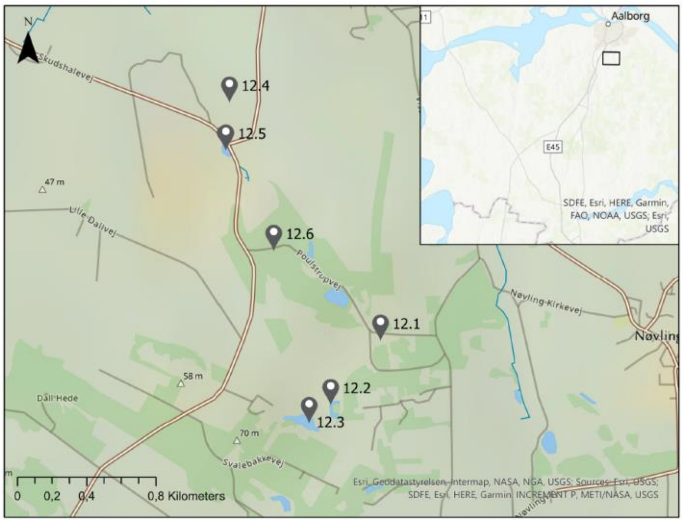

Figure A23. Area 12. The area of investigation consists of two sites, both surrounded by agricultural fields and close to buildings.

12.1 is 900 m^2 and <0.7 m deep. Aquatic vegetation such as Juncaceae covers 95% of the water table. Medium vegetation (16–25 cm) and trees (*Betula*) dominate the shoreline. The site is surrounded by heathland and forest grazed extensively by sheep and cattle.

12.2 is a part of a large wetland area of which 800 m^2 is searched for amphibians. It has a depth of <1.3 m, and aquatic vegetation such as Juncaceae covers 85% of the water table. Medium to high vegetation (16–50 cm), trees, and bushes dominate the shoreline. The site is surrounded by heathland and meadow. *R. arvalis* and *R. temporaria* were observed at the site at the time of sampling.

12.3 is a part of a large lake of which 400 m^2 is searched for amphibians. The searched area has a depth of < 1.4 m, and aquatic vegetation covers 90% of the water table. High vegetation (26–50 cm), mostly *Juncus effusus* and trees, dominates the shoreline. *R. temporaria* and *B. bufo* were observed at the site at the time of sampling. The site is surrounded by heathland and meadow

12.4 is 430 m^2 and <1.2 m deep. Aquatic herbs cover 20% of the water table, and medium to high vegetation (16–50 cm) such as *Juncus effusus* and Poaceae dominates the shoreline. The site is in a clearing surrounded by a pinewood forest.

12.5 is 2000 m^2 and 70% is < 1.2 m deep. Submerged vegetation covers 95% of the water table. Medium vegetation (16–25 cm) and overgrowth (>50 cm) by *Phragmites australis* dominate the shoreline. The site is in a small village with buildings and roads nearby.

12.6 is 650 m^2 and <0.5 m deep. Aquatic vegetation such as *Equisetum* and *Juncus* cover 95% of the water table. High vegetation (26–50 cm) and trees (*Betula*) dominate the shoreline. The site is surrounded by grassland grazed by cattle and sheep.

Table A12. Overview of species findings by conventional methods in previous study and eDNA metabarcoding. – indicates no findings. The previous study used as reference is in this instance is Neergaard [2].

Site	Present Study	Previous Study
	**eDNA Metabarcoding**	**Conventional**
12.1	-	*Rana temporaria*
12.2	*Lissotriton vulgaris*<br>*Rana temporaria*	*Lissotriton vulgaris*<br>*Rana arvalis*
12.3	*Lissotriton vulgaris*	*Triturus cristatus*<br>*Rana arvalis*<br>*Rana temporaria*

		Bufo bufo
12.4	*Lissotriton vulgaris*<br>*Triturus cristatus*	*Triturus cristatus*<br>*Rana temporaria*
12.5	*Lissotriton vulgaris*<br>*Rana temporaria*	*Triturus cristatus*<br>*Rana arvalis*<br>*Rana temporaria*<br>*Bufo bufo*
12.6	*Lissotriton vulgaris*<br>*Triturus cristatus*<br>*Bufo bufo*	*Rana arvalis*<br>*Rana temporaria*<br>*Bufo bufo*

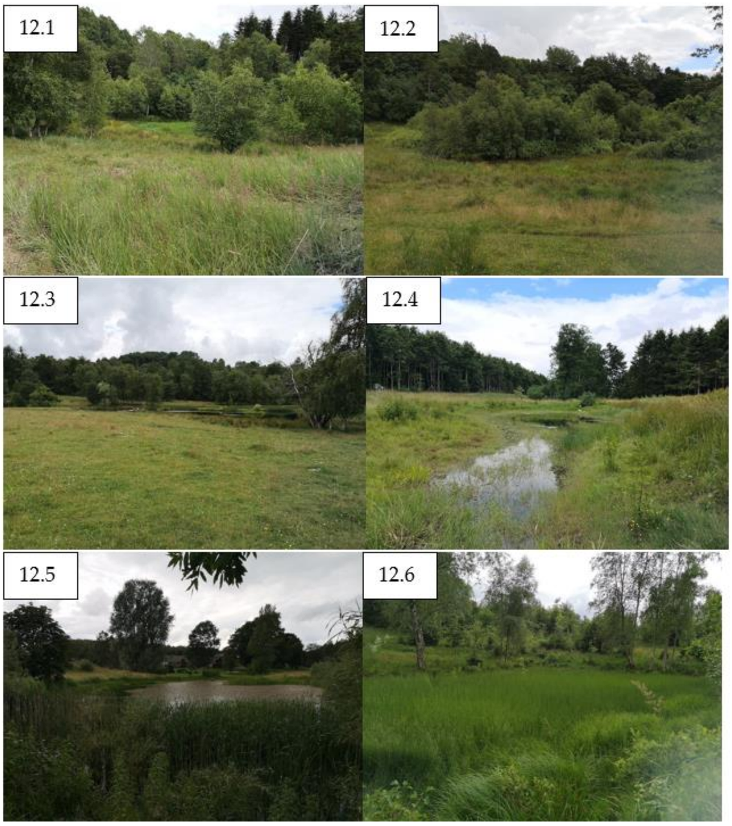

Figure A24. Photos of the six sites constituting area 12.

Supplementary B—Scheme for Assessment of Amphibian Habitats

The following scheme was developed on the basis of a similar scheme for monitoring of Annex II species at the Habitat directive [3] as well as a point system. It was included in a semester project during the 2. semester on Master's in Biology at Aalborg University by Skrubbeltrang and Svenningsen [43].

Assessment of amphibian habitats

**Date:**		**Site:**	
**Coordinates:**	Lat: Long	**Time:**	

1: Edaphic conditions

Moisture (mark by x):		Standing water (mark by x):		Edaphic type (mark by x):			
Yes:	No:	Yes:	No:	Sand:	Gravel:	Loose soil:	compressed soil:

2: If "yes" to "standing water", answer question 3-7. If "no", answer question 8.

3: Drying out (mark by x):	Often:	Sometimes:	Rarely:	Never:

4: Covorage of aquatic plants (mark by x):

0%:	1-5%:	6-25%:	26-50%:	51-75%:	76-100%:

Notes (e.g. algae, benthic plants, submerged plants ...)

If plants are made up of flooded terrestrial species, please note it her.

5: Growth on shoreline (specify coverage ranging from 1-7 to the right)

0% (1)	1-5% (2)	6-25% (3)	26-50% (4)	51-75% (5)	76-100% (6)	**Specify 1-7:**
No vegetation (0 cm)						
Low vegetation (1-15 cm)						
Medium vegetation (16-25 cm)						
High vegetation (26-50 cm)						
Overgrown(>50 cm)						

6: Part of shoreline with caltivated fields (if the location consists of multiple ponds, please state the percentage of the accumulated covered shoreline).

0%:	1-5%:	6-25%:	26-50%:	51-75%:	76-100%:

7: Part of shoreline with shade (if the location consists of multiple ponds, please state the percentage of the accumulated shaded shoreline).

0%:	1-5%:	6-25%:	26-50%:	51-75%:	76-100%:

8: Surrounding nature (nature outside the location) (please, indicate multiple with x):

Forest		Open without trees/bushes		Sandy dunes	
Dense growth		Open with trees/bushes		Bog	
Wet lands/lakes		Wit no/little vegetation		Agricultural fields	

Notes on surrounding nature:

9: Growth on the location (specify coverage from 1-7 to the right)

0% (1)	1-5% (2)	6-25% (3)	26-50% (4)	51-75% (5)	76-100% (6)	Specify 1-7:
No vegetation (0 cm)						
Low vegetation (1-15 cm)						
Medium vegetation (16-40 cm)						
High vegetation (41-75 cm)						
Overgrown (>75 cm)						

10: Characteristics of the area (please, indicate multiple with x):					
Sign on management (specify ↓)		Ditches		Ponds	
Trafic <250 m		Settlements		Shallow parts	
Trafic >250 m		Fishes, crayfish, waterfowl		Fragmentation	
Slopes or enbankments		In and outlets		Streams (to/from)	
Notes on characteristics:					

11: Distance to nearest suitable pond (mark by x):							
<10 m	<50 m	<100 m	<200 m	<500 m	<1 km	<2 km	>2 km

12: Distance to next suitable pond (mark by x):							
<10 m	<50 m	<100 m	<200 m	<500 m	<1 km	<2 km	>2 km

13: Amphibians observed (mark by x):		Notes on amphibians (species, song, eggs...):
Yes:	No:	
Further comments:		

Aditional information on abiotic factors						
Information in this paragraph is not included in scorring system						
If "yes" to "standing water", please answer a, b and c. If "no", please answer d.						
a: Water depth:	m	c: turbidity: (mark by x)	Clear	Blurry	Turbid	Dark
b: Water temperature	C					
d: Wheather conditions	Wind:	Temp:	Cloud cover:	Lee:		
	m/s	C		Yes:	No:	

Figure 1. Scheme for assessment of habitat structures important for amphibian presence. In [43] are point system of the system elaborated.

Supplementary C—Filtered water

The amount of filtered water of each site when colleting samples for eDNA metabarcoding are listed in Table D1 as well as comments of observation from the time of sampling. All observations of juvenile and adult amphibians were on land.

The average amount of filtered water was below average for NatureMetrics filters. This indicates substantial amounts of particles clotting the filters, naturally limiting the amount of DNA filtered and potentially affecting species detection. Filters with larger pore size should be considered if sampling on sites with large amounts of suspended materials. A pore size of 1–10 μm has been shown to capture the majority of DNA from macroorganisms.

Table 1. The amount of filtered water per site (mL) in collection of eDNA for metabarcoding analysis. Relevant comments of observations at the time of sampling are also noted.

	Filtered Water (mL)	Comments
1.1	690	
1.2	170	Multiple juvenile *Rana arvalis*
1.3	530	
1.4	680	12 subsamples due to overgrowth at shoreline

2.1	790	
2.2	20	Suspended bottom material
2.3	190	
2.4	200	
3.1	20	Suspended bottom material
4.1	1150	

Supplementary D—Rare fraction curve

Rarefaction curve of ZOTU's showing flattening curves as the number of reads increases indicating that the majority of the diversity of the samples have been sequenced.

Table 1. continued:.

	Filtered Water (mL)	Comments
4.2	830	
4.3	70	Suspended bottom material
4.4	520	
5.1	360	One adult of *Rana* species
5.2	270	Tadpole of *Rana temporaria*
5.3	530	
6.1	530	
6.2	550	Juvenile of *Rana temporaria*
6.3	780	
7.1	280	
8.1	180	Large amounts of suspended organic material
8.2	800	
8.3	400	Large degree of evaporation since conventional survey
8.4	450	Multiple juveniles of *Rana* species and adult of *Triturus cristatus*
8.5	800	
9.1	130	Large degree of evaporation since conventional survey
9.2	540	
10.1	430	
10.2	170	Large amounts of suspended organic material
10.3	100	Large amounts of suspended organic material
10.4	240	
10.5	140	Large amounts of suspended organic material
10.6	300	Multiple juvenile *Bufo bufo*
10.7	960	Multiple juvenile *Bufo bufo*
10.8	230	
11.1	110	Large amounts of suspended organic material
11.2	200	
11.3	150	Large amounts of suspended organic material
11.4	200	
11.5	250	Multiple juveniles of *Rana* species and *Bufo bufo*
11.6	250	
12.1	570	Multiple juvenile *Bufo bufo* and adult *Lissotriton vulgaris*

12.2	320	Multiple juveniles of *Rana* species
12.3	270	Multiple juveniles of *Rana* species and adult *Byfo bufo*
12.4	1490	Multiple juvenile *Bufo bufo*
12.5	1060	Multiple juvenile *Bufo bufo*
12.6	420	

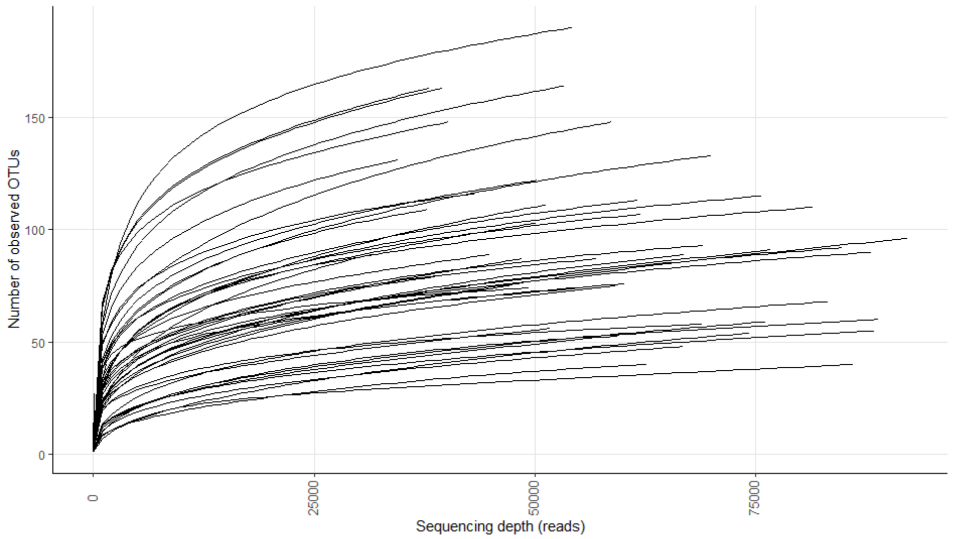

Figure 1. Rarefaction curve of sequenced ZOTUs with the sequencing depth in number of reads on the x-axis and the number of observed ZOTUs on the y-axis.

Supplementary E—Additional Results

The relative frequency of occurrence for all species was calculated to compare the species detection across survey methods applied in the present and previous studies (table E1) [16, 17].

Table 1. The relative frequency of occurrence in percent (%) for detected species per survey method. The survey methods applied in the present study are eDNA metabarcoding and conventional methods, while previous studies applied conventional surveys exclusively.

Expected Species	eDNA Metabarcoding	Present Conventional Survey
Lissotriton vulgaris (Smooth newt)	43	20
Triturus cristatus (Great crested newt)	19	17
Rana arvalis (Moor frog)	7	28
Rana temporaria (Common frog)	17	20
Bufo bufo (Common toad)	14	9
Epidalea calamita (Natterjack toad)	0	7
Pelobates fuscus (Common spade-foot toad)	0	2

In the calculation of the Sørensens similarity index, values in Table E2 are applied. The table gives an overview of unique and overlapping observations of eDNA metabarcoding and conventional survey methods applied in the present study for each expected species, as well as the index value.

Table 2. Overview of unique and overlapping observations of eDNA metabarcoding and conventional survey methods applied in the present study for each expected species. The index value of the Sørensens Similarity Index also appears.

**Expected species**	**Unique Observation by eDNA Metabarcoding Survey**	**Unique Observations for Conventional Survey**	**Overlapping Observations**	**Index Value**
Lissotriton vulgaris (Smooth newt)	9	9	6	0.40
Triturus cristatus (Great crested newt)	5	9	3	0.30
Rana arvalis (Moor frog)	2	13	1	0.12
Rana temporaria (Common frog)	4	9	2	0.24
Bufo bufo (Common toad)	3	4	1	0.22
Epidalea calamita (Natterjack toad)	0	3	0	0.00
Pelobates fuscus (Common spade-foot toad)	0	0	0	0.00
